# Lamin A upregulation reorganizes the genome during rod photoreceptor degeneration

**DOI:** 10.1038/s41419-023-06224-x

**Published:** 2023-10-25

**Authors:** Ivana Herrera, José Alex Lourenço Fernandes, Khatereh Shir-Mohammadi, Jasmine Levesque, Pierre Mattar

**Affiliations:** 1 Ottawa Hospital Research Institute (OHRI), Ottawa, ON K1H 8L6 Canada; 2 Department of Cellular and Molecular Medicine, University of Ottawa, Ottawa, ON K1H 8M5 Canada

**Keywords:** Cell death in the nervous system, Transcriptomics

## Abstract

Neurodegenerative diseases are accompanied by dynamic changes in gene expression, including the upregulation of hallmark stress-responsive genes. While the transcriptional pathways that impart adaptive and maladaptive gene expression signatures have been the focus of intense study, the role of higher order nuclear organization in this process is less clear. Here, we examine the role of the nuclear lamina in genome organization during the degeneration of rod photoreceptors. Two proteins had previously been shown to be necessary and sufficient to tether heterochromatin at the nuclear envelope. The lamin B receptor (Lbr) is expressed during development, but downregulates upon rod differentiation. A second tether is the intermediate filament lamin A (LA), which is not normally expressed in murine rods. Here, we show that in the *rd1* model of retinitis pigmentosa, LA ectopically upregulates in rod photoreceptors at the onset of degeneration. LA upregulation correlated with increased heterochromatin tethering at the nuclear periphery in *rd1* rods, suggesting that LA reorganizes the nucleus. To determine how heterochromatin tethering affects the genome, we used in vivo electroporation to misexpress LA or Lbr in mature rods in the absence of degeneration, resulting in the restoration of conventional nuclear architecture. Using scRNA-seq, we show that reorganizing the nucleus via LA/Lbr misexpression has relatively minor effects on rod gene expression. Next, using ATAC-seq, we show that LA and Lbr both lead to marked increases in genome accessibility. Novel ATAC-seq peaks tended to be associated with stress-responsive genes. Together, our data reveal that heterochromatin tethers have a global effect on genome accessibility, and suggest that heterochromatin tethering primes the photoreceptor genome to respond to stress.

## Introduction

Photoreceptor cells are highly susceptible to degeneration—perhaps due to their very high metabolic demands [1]. Cone photoreceptors are responsible for high-acuity color vision, whereas rod photoreceptors mediate vision in low-light conditions. Indeed, rods are sensitive enough to respond to individual photons [2]. To achieve this feat, rods must maintain high-level expression of at least 50 genes that can lead to degeneration when misregulated [3, 4]. Genome regulation is thus essential for photoreceptor survival.

The importance of genome organization in photoreceptors is further underscored by their specialized nuclear architecture. In mice, rods undergo a process called “chromatin inversion” [5–7]. Whereas most cells tether heterochromatin in ‘lamina-associated domains’ at the nuclear periphery, rod photoreceptors localize heterochromatin centrally [5–8]. Throughout mammalian evolution, chromatin inversion is correlated with nocturnal lifestyle, as the inverted configuration decreases light scattering and enhances contrast sensitivity [7, 9]. At the molecular level, two proteins have been shown to be sufficient for heterochromatin tethering.

1) The lamin B receptor (Lbr) is a multi-pass transmembrane receptor that contains an intra-nuclear tudor domain. Murine rods naturally express Lbr during development, although Lbr levels decline once rods differentiate. However, when Lbr expression in rods was artificially sustained, chromatin inversion was prevented [10].

2) The *Lmna* gene encodes two splice variants—lamin A (LA) and lamin C (LC), neither of which is normally expressed in murine rod photoreceptors [10–13]. These A-type lamins are intermediate filaments that form a meshwork across the surface of the inner nuclear membrane. In *Lmna* knockout mice, heterochromatin tethering was lost in various tissues, but transgenic misexpression of LC in rods had no effect on their inverted organization [10], initially suggesting that A-type lamins were *not* sufficient for heterochromatin tethering. However, we showed that LA is sufficient to tether heterochromatin in rods [12], resolving this conundrum. Interestingly, the unique LA C-terminus was recently shown to interact with histone H3, while the equivalent domain of LC cannot [14], which potentially explains this functional divergence.

Interestingly, degenerating rods were found to exhibit altered nuclear organization in a variety of mice harboring mutations in chromatin proteins [10, 12, 15–19]. While these data potentially link the associated chromatin proteins to nuclear architecture, the observed alterations might instead be driven by cell death. Alternatively, chromatin proteins might act indirectly by altering gene expression. For example, mutants for the *Nrl* and *Nr2e3* transcription factors lead to a rod-to-cone fate switch that reorganizes the nucleus [20, 21]. In addition, A-type lamins have been shown to upregulate in some knockouts [10, 12, 16], raising the possibility that heterochromatin tethers might contribute to degeneration-associated nuclear reorganization.

Here, we show that LA upregulates in the *rd1* mouse—one of the best studied models for retinitis pigmentosa. Rod degeneration in the *rd1* mutant is triggered via toxic accumulation of cyclic guanosine monophosphate—a chromatin-independent process. Using genomic and transcriptomic approaches, we find that heterochromatin tethering may help to reconfigure the genome to respond to environmental insults.

## Results

### Lamin A upregulates during rod photoreceptor degeneration

**Fig. 1:**
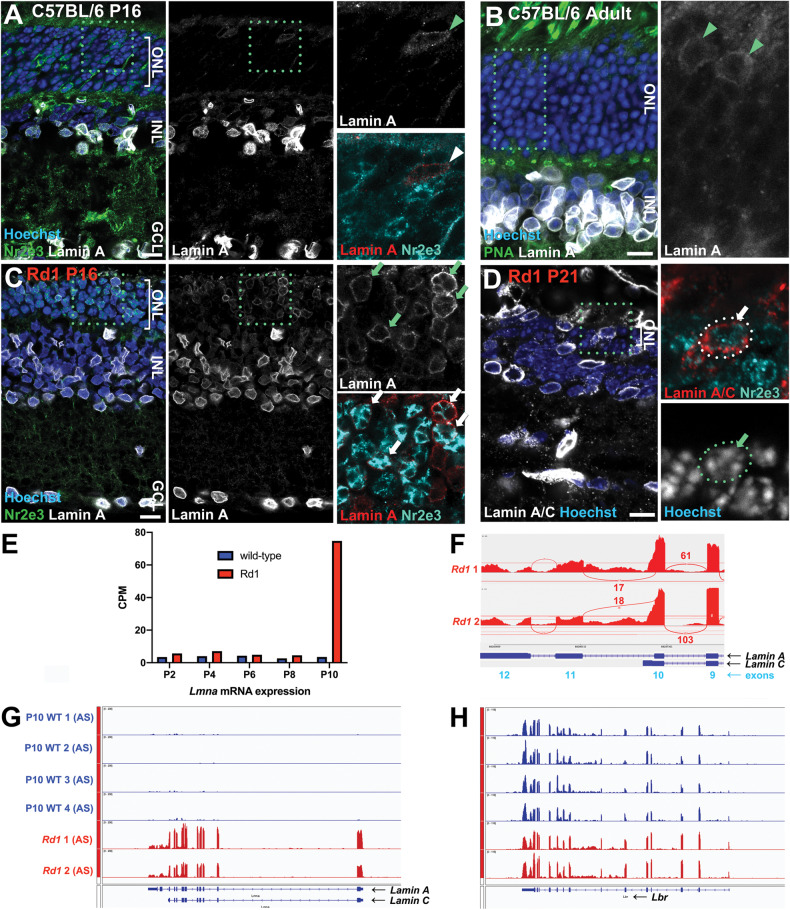
Lamin A upregulates at the onset of rod degeneration in *rd1* rods. **A**, **B** Immunohistochemistry on wild-type C57BL/6J at P16 (**A**) or adult (**B**) stages using a LA-specific antibody (white). The retina was counterstained for the rod marker Nr2e3 (**A**; green) or the cone marker peanut agglutinin (**B**; green), as well as the DNA dye Hoechst 33342 (blue). Boxed regions indicate the areas shown in the insets. Arrowheads indicate cone photoreceptors. **C**, **D** Immunohistochemistry for LA or LA/LC (white) and the rod-specific marker Nr2e3 (green) on *rd1* retinas at P16 (C) or P21 (**D**). Boxed regions indicate the areas shown in the insets. Arrows indicate LA expression in Nr2e3+ rods. Scale bars = 10 µm. **E**–**H** Transcript expression at different postnatal stages as indicated—from the Anand Swaroop lab (AS) [24]. **F** Sashimi plot of splice junctions from P10 *rd1* RNA-seq data. **G**, **H** Transcription at the *Lmna* (**G**) or *Lbr* (**H**) loci from P10 RNA-seq samples as indicated. Data were re-mapped from Jiang et al. [24] and plotted on the same scale (group autoscale).

LA is sufficient to reorganize the rod nucleus [12], whereas LC has no effect [10]. Using an isoform-specific antibody (Fig. S1A), we found that in wild-type mice, LA was extensively expressed within the inner retina (Fig. 1A, B, Supplemental video 1), as reported previously [13]. In photoreceptors, LA immunoreactivity was observed only in cones (Fig. 1A, B, arrowheads), and was absent from rods (Fig. 1A, B). *Lmna* transcription was also little detected in chick, human, or macaque rods as determined via the *Plae* scRNA-seq database [22] (Fig. S1B), in accordance with previous studies [10].

Previous studies revealed nuclear disorganization and LA/LC upregulation in mice harboring mutations in essential chromatin proteins (e.g., Casz1, Atxn7), but whether this was a general feature of degeneration remained unclear. To address this question, we examined *rd1* mice—a well-studied degenerative model in which rods are completely eliminated by the fourth postnatal week. The *rd1* mutation disrupts the *Pde6b* gene, which is linked to retinitis pigmentosa in humans [23]. Examination of P16 *rd1* mice revealed extensive LA expression within the degenerating photoreceptor layer (Fig. 1C). Since cone cell death is more protracted versus rods in the *rd1* model, we co-stained *rd1* retinas with the rod-specific transcription factor Nr2e3 (Fig. 1C, D, Supplemental video 2). This confirmed that LA upregulated in bona fide rods in the *rd1* retina.

To corroborate these observations, we examined a recently published RNA-seq dataset made from sorted *rd1* rods [24]. In this dataset, *Lmna* was among the most significantly upregulated genes. Moreover, *Lmna* upregulation coincided precisely with the onset of cell death at P10 (Fig. 1E). Since pathological gene expression begins as early as P2 [24], these data argue that *Lmna* upregulates in response to degeneration. To visualize *Lmna* transcripts directly, we re-mapped Jiang et al.’s RNA-seq data. *Lmna* upregulated significantly in the reanalyzed data (Log_2_ fold-change vs. control: 3.40; adj. *p*-value: 5.19E−13). Examination of *Lmna* exon usage revealed that alternative splicing generated bona fide *LA* transcripts (Fig. 1F, G). Finally, we examined *Lbr* transcripts and protein, but found no difference in wild-type versus *rd1* mice (Log_2_ fold-change vs. control: 0.042; adj. *p*-value: 0.91; Fig. 1H; Fig. S2). Thus, degenerating rods upregulate LA, raising the possibility that higher-order genome organization might be reconfigured in these photoreceptors.

**Fig. 2:**
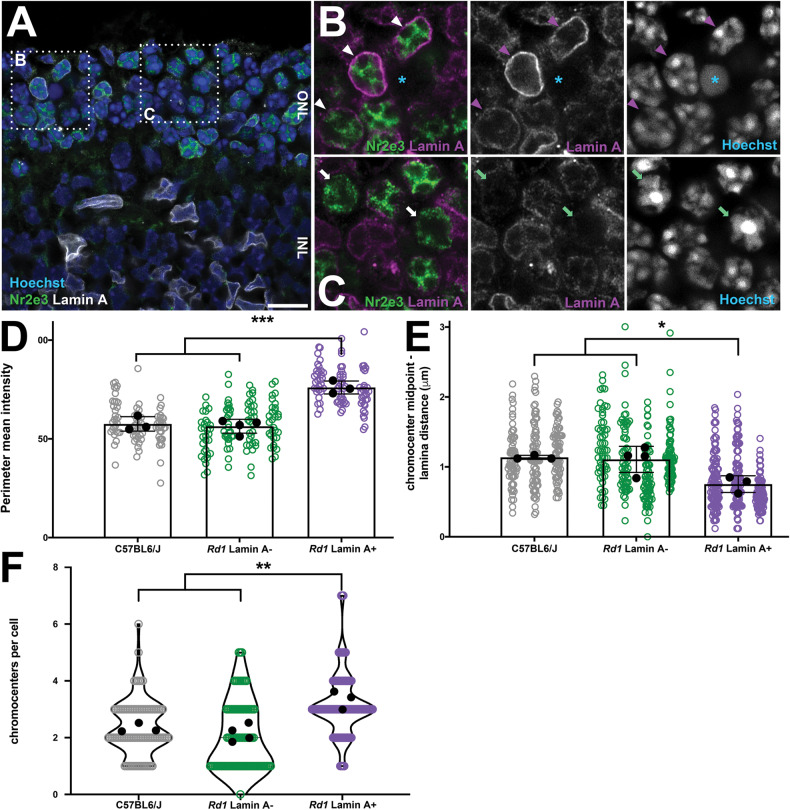
Increased heterochromatin tethering in Lamin A+ *rd1* rods. **A** Airyscan confocal imaging of P16 *rd1* retinas stained for LA (white), Nr2e3 (green), and Hoechst (blue). Boxed regions indicate the areas shown in (**B**, **C**). Arrowheads indicate LA+ Nr2e3+ rods. Arrows indicate LA-negative Nr2e3+ rods. Asterisk indicates a pyknotic nucleus. Scale bars = 10 µm. **D** Mean chromatin intensity at the nuclear margin normalized against the mean chromatin intensity of the whole nucleus. **E** Linear distance between chromocenter centroids and the nuclear margin. **F** Chromocenters per nucleus. Black datapoints and error bars are the mean intensity values from each biological replicate (30 cells each; circles) ± SEM. *p < 0.05, **p < 0.01, ***p < 0.001; one-way ANOVA with Tukey’s post-hoc test.

To test this idea directly, we measured heterochromatin tethering in LA-positive versus -negative rods. We found that the intensity of DNA at the nuclear periphery was elevated when LA+ cells were compared to LA-negative cells from the *rd1* mouse, or to C57BL6/J controls (Fig. 2A–D). Similarly, the distance between each chromocenter and the margin of the nucleus was significantly reduced in LA+ versus C57BL6/J control rods (Fig. 2E), suggesting that LA reorganizes the nucleus during degeneration.

### Heterochromatin tethering by Lamin A versus Lbr

**Fig. 3:**
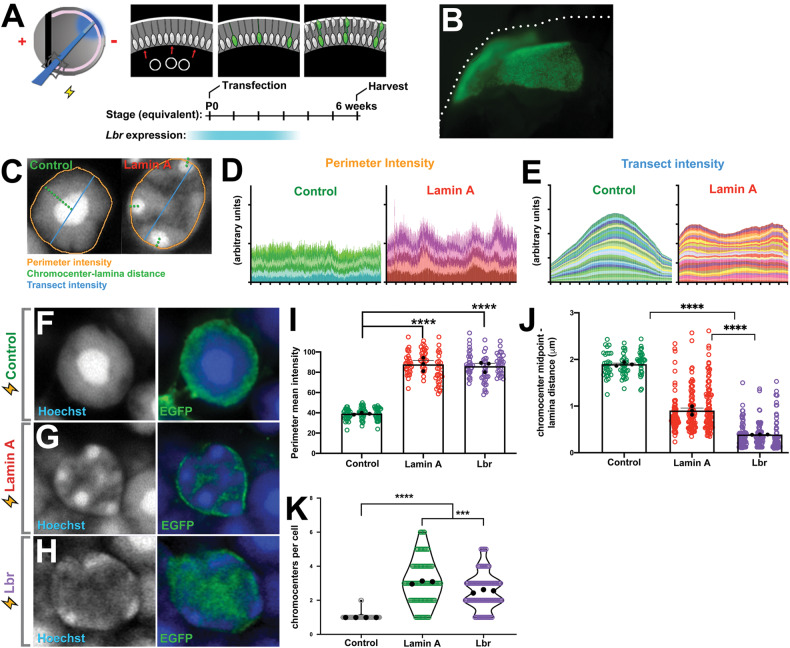
Heterochromatin tethering by lamin A versus Lbr. **A** In vivo electroporation paradigm. Retinas were subretinally injected with plasmids, electroporated, and harvested after 6 weeks, yielding transfected rod photoreceptors. **B** Wholemount epifluorescence image of EGFP expression from a transfected retina. **C** Morphometric analysis of transfected nuclei was performed as indicated. **D** Densitometry values for chromatin intensity at the nuclear periphery of Control (*n* = 6) or LA-transfected cells (*n* = 6) as indicated, obtained using the “Plot profile” tool in Fiji. Each color represents a different cell. **E** Densitometry values for chromatin intensity transecting the center of the nucleus from Control (*n* = 30) or LA-transfected cells (*n* = 30). **F**–**H** Airyscan confocal imaging of rod photoreceptors transfected with GFP-expressing empty vector control (**F**), LA (**G**), or Lbr (**H**) constructs, and harvested after 6 weeks. **I** Quantitation of chromatin intensity at the nuclear margin measured using the “Freehand Line” tool in Fiji. **J** Distance from the chromocenter midpoint to the nuclear periphery. **K** Chromocenter number per cell. Black datapoints and error bars are the mean intensity values from each biological replicate (30 cells each; circles) ± SEM. *p* < 0.0001, one-way ANOVA with Tukey’s post-hoc test. ***p < 0.001, ****p < 0.0001, ANOVA with Tukey’s post-hoc test.

While LA+ rods exhibited increased heterochromatin tethering, *Lbr* is still expressed when LA upregulates (Fig. 1G, H) raising the question of whether LA and Lbr tether heterochromatin differently. To compare LA versus Lbr-dependent heterochromatin tethering, wild-type retinas were electroporated at P0 with control, LA, or Lbr expression constructs cloned into the pCIG2 vector, which contains an IRES2-EGFP reporter cassette. Importantly, due to the exclusively embryonic temporal window for cone generation, cone photoreceptors are never transfected [25–27]. Transfected rods were examined at P42, when chromatin inversion is complete (Fig. 3A).

Using morphometric measures for heterochromatin tethering (Fig. 3C–E), we found that both LA and Lbr increased the intensity of DNA at the nuclear periphery equivalently in comparison to control cells (Fig. 3F–I; Fig. S3). Chromatin inversion progressively reduces the number of chromocenters [7]. In Lbr-transfected cells, the distance between the chromocenter centroid and nuclear lamina was significantly reduced in rods expressing Lbr versus LA (Fig. 3J). Moreover, LA increased chromocenter number significantly more than Lbr (Fig. 3K). LA and Lbr thus had different effects on nuclear organization. In accordance with these observations, we found no evidence of cross-regulation. Lbr did not upregulate in LA transfections, nor did LA upregulate in Lbr transfections (Fig. S4).

### Heterochromatin tethers have subtle effects on gene expression

**Fig. 4:**
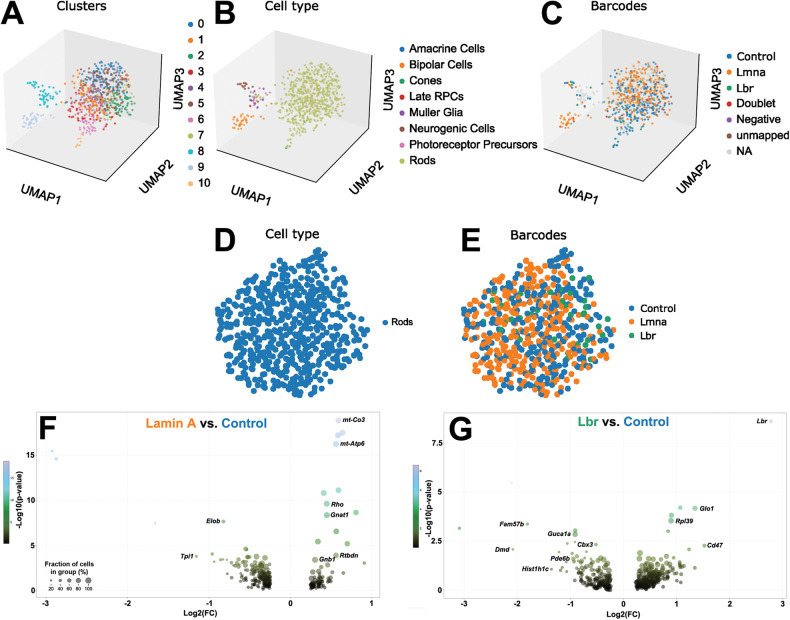
Comparison of gene expression in control versus tethered rods. **A** UMAP projection of Multi-seq dataset. **B** Unsupervised cell-type annotation via scDeepSort trained on a previously published retinal scRNA-seq dataset [29]. **C** Demultiplexing of control, lamin A, and Lbr samples. **D**, **E** Overlap of Control, lamin A, or Lbr-transfected cells within the rod cluster. **F**, **G** Volcano plots of differential gene expression in annotated rods. **F** Lamin A versus control. **G** Lbr versus control.

To determine how tethering affects gene expression, we misexpressed LA/Lbr in rods using in vivo electroporation. After 8 weeks, we flow-sorted viable EGFP+ cells. As we were only able to obtain a few thousand cells per transfected retina, we opted to perform scRNA-seq using the 10x Genomics Chromium platform. To avoid batch effects, we multiplexed samples using the Multi-seq barcoding approach. After removing low-quality cells and performing additional filtering (see Methods), we clustered individual cells using Uniform Manifold Approximation and Projection (UMAP; Fig. 4A). To annotate cell types in an unsupervised manner, we used scDeepSort [28] trained on a previously published retinal RNA-seq atlas [29]. As expected, most sorted cells in the dataset were rods, but a few bipolars and Müllers were also annotated (Fig. 4B).

Focusing solely on annotated rods, Control, LA, and Lbr-transfected cells clustered in an overlapping fashion, suggesting little difference between their overall gene expression patterns (Fig. 4D, E). Similarly, we found that both LA and Lbr had relatively modest effects on the expression of individual genes (Fig. 4F, G; Table S1). Characteristic photoreceptor genes were significantly elevated in LA-expressing rods (e.g., *Rho*, *Gnat1*; Fig. 4F), and significantly decreased in Lbr-expressing cells (e.g., *Guca1a*, *Pde6b*; Fig. 4G). Nonetheless, the overall magnitude of transcriptional changes in tethered rods was generally modest, with only a few genes changing more than 2-fold. Interestingly, *Hist1h1c* and *Cbx3*, which encode key heterochromatic proteins, were significantly downregulated in Lbr-expressing rods (Fig. 4G).

### Heterochromatin tethering regulates genome accessibility

**Fig. 5:**
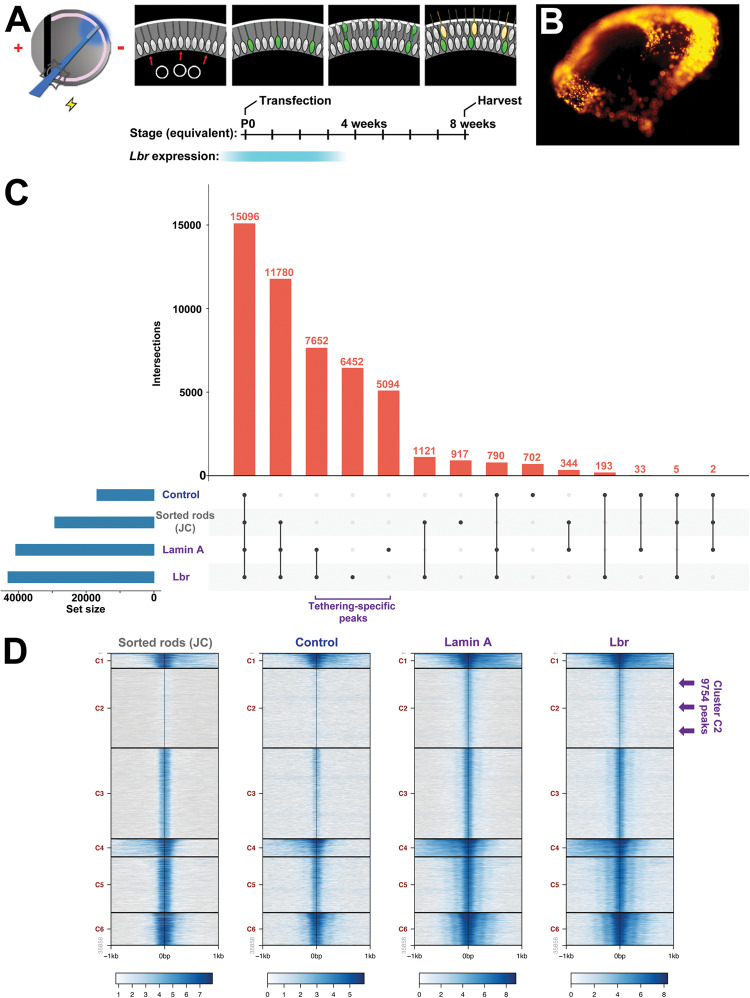
Heterochromatin tethering promotes chromatin accessibility. **A** In vivo electroporation paradigm. Retinas were subretinally injected with expression plasmids, including the rod-specific pRho2.2-DsRed reporter. Electroporated retinas were harvested after 8 weeks, yielding DsRed+ rod photoreceptors. **B** Wholemount epifluorescence image of EGFP and DsRed expression from a transfected retina. **C** Upset plot of ATAC-seq peak intersections from sorted rods transfected with control, LA, or Lbr expression constructs compared against previously published data from the Joe Corbo lab (JC) [11] as indicated. **D** Alignment of ATAC-seq data from sorted rods transfected with control, LA, or Lbr expression constructs compared against previously published data [11] as indicated. Plots are centered on peak summits from LA transfected rods. Arrows indicate the tethering-specific cluster C2 peaks.

Previous studies have reported that rod photoreceptors uniquely exhibit megabase-scale genomic intervals with unusually reduced chromatin accessibility [11]. We reasoned that this unique accessibility signature might be altered by heterochromatin tethering. We therefore performed ATAC-seq on rods transfected with control, LA, or Lbr constructs. To mark rods specifically, we co-transfected the plasmids with a pRho2.2::DsRed reporter [30] (Fig. 5A, B). After 8 weeks, rod photoreceptors were sorted using EGFP, DsRed, and Dapi to monitor viability.

Next, we processed the ATAC-seq datasets in order to call peaks. We first compared our datasets against previously published ATAC-seq data from sorted rods [11]. In general, we observed that all of our datasets exhibited comparable signal at the rod-specific peak loci previously identified by Hughes et al. [11] (Fig. S5). We also observed a lack of signal at the promoters of several marker genes for non-rod cell types, such as cones, bipolars, and Müller glia (Fig. S6).

In accordance with the hypothesis that inverted nuclear architecture restricts accessibility [11], we observed an increase in the number of peaks in tethered rods (Fig. 5C). We compared LA or Lbr-tethered rod ATAC-seq data versus normal rod datasets using two approaches. First, we examined the overlap between peaks. We found that more than half of the rod-specific peaks identified by Hughes et al. were shared by our ATAC-seq datasets (Fig. 5C). The control datasets together contained less than 2000 peaks that were absent from tethered rods. By contrast, LA and Lbr datasets exhibited 5094 and 6452 unique peaks, respectively, and shared an additional 7652 peaks—all of which were absent from the control datasets (Fig. 5C).

Secondly, we also plotted the data centered on the 35 858 LA peaks. This analysis revealed a great deal of resemblance between the accessibility signatures of LA+ and Lbr+ rods. We performed K-means clustering which allowed us to separate 9754 peaks that had markedly elevated signal in LA/Lbr tethered rods in comparison to controls (Fig. 5D; cluster C2; arrows). Peak-to-gene annotation revealed that only ~10% of these novel peaks were found in gene-proximal regions (Fig. S7A). Taken together, these analyses indicate that rods with tethered heterochromatin gain thousands of additional peaks—mainly in distal intergenic regions.

We inspected newly accessible peaks, but they did not appear to overlap with any specific genomic feature. We therefore opted to perform footprinting analysis using the TOBIAS algorithm [31]. TOBIAS examines ATAC-seq peaks to identify regions occluded by proteins, and to match these ‘footprints’ to transcription factor motifs. We selected 220 transcription factor motifs from the TRANSFAC database. Using this approach, we found that Ctcf footprints were the most overrepresented motifs in both LA and Lbr datasets (Fig. S7B, C). Focusing on novel tethering-specific cluster C2 peaks, we next visualized Ctcf ChIP-seq datasets made from embryonic stem cells by the Bing Ren lab [32]. We found that cluster C2 loci correlated with considerable Ctcf signal (Fig. S7D). These data suggest that many of the peaks induced by heterochromatin tethering are genuine regulatory elements that are normally decommissioned in rods. Perhaps accordingly, we found that cluster C2 loci exhibited no obvious enrichment for chromatin marks from published rod-specific datasets [8, 33] (Fig. S8).

Hughes et al. had also previously hypothesized that heterochromatin tethering might explain the increased genomic accessibility of cones [11]. However, we found that in terms of genome accessibility, tethered rods are more similar to control rods than to cones—perhaps not surprisingly (Fig. S9A). Inspection of cone-specific peaks revealed that most remained inaccessible in tethered rods (Fig. S9B; see also Fig. S6), except at the *Lmna* locus itself (Fig. S9C), which was previously noted to be accessible in cones but not rods [11]. Some loci might therefore take on cone-like accessibility signatures in response to tethering.

Finally, to address the hypothesis that heterochromatin tethering might affect the accessibility of B compartment topologically associating domains (TADs), we examined rod-specific Hi-C experiments [16]. Surprisingly, we found that almost all of the novel cluster C2 peaks were present in the euchromatic A compartment (Fig. S10A). We did observe a few notable exceptions, where B compartment accessibility was altered, including at the *Myc* gene (Fig. S10B), which was previously reported to be localized within a large inaccessible interval [11], as well as across a TAD that contains the chemokines *Ccl1*, *Ccl2*, *Ccl7*, *Ccl8*, *Ccl11*, and *Ccl12* (Fig. S10C). Nonetheless, effects on B compartment accessibility were the exception.

### Heterochromatin tethering promotes accessibility at a subset of stress-responsive genes

**Fig. 6:**
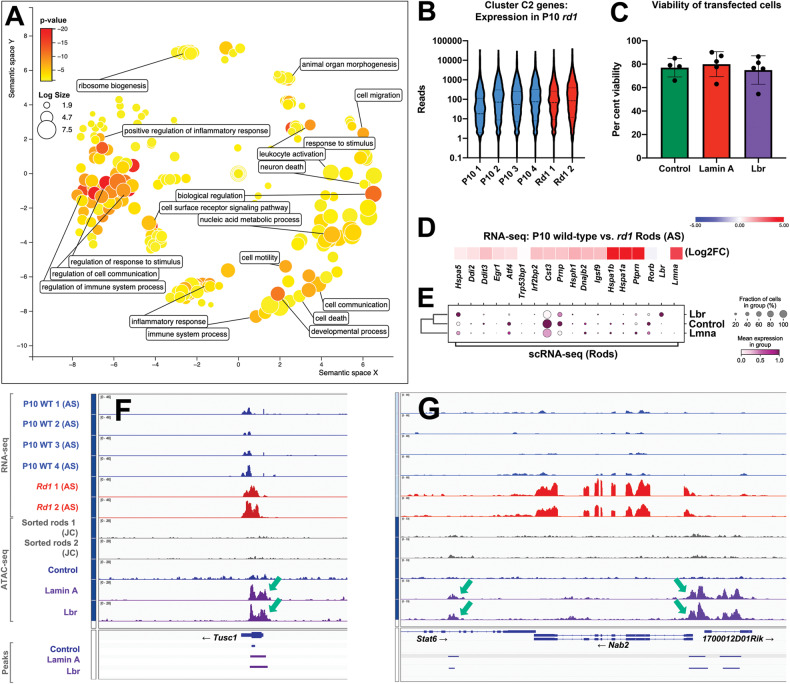
Heterochromatin tethering promotes accessibility at stress-responsive genes. **A** GO terms analysis of tethering-specific cluster C2 genes (see Fig. 4D) via PantherDB and ReViGO. Peak-to-gene annotation was restricted to gene proximal peaks as described in the text. **B** Violin plot of mRNA expression for genes associated with cluster C2 peaks from P10 control or *rd1* sorted rods. RNA-seq data were generated by the Swaroop lab [24]. **C** Cell viability data from transfected (GFP+/DsRed+) cells harvested after 8 weeks, based on Dapi exclusion (not significantly different via one-way ANOVA). **D**, **E** Expression changes for selected stress-responsive genes, comparing RNA-seq data from control versus *rd1* sorted rods from the Anand Swaroop lab (AS) [24] (**D**) versus scRNA-seq data from rods transfected with control, lamin A, or Lbr plasmids (**E**). **F**, **G** Control vs. *rd1* RNA-seq, and ATAC-seq tracks and called peaks from Hughes et al. (JC) [11], compared against ATAC-seq tracks generated from control, LA, or Lbr-transfected rods at the *Tusc1* (**F**) and *Nab2* (**G**) loci. RNA-seq and ATAC-seq tracks were respectively group-autoscaled. Arrows indicate peaks present specifically in tethered rods, but not control rods.

To understand how heterochromatin tethering might relate to function, we performed GO terms analysis on the tethering-specific cluster C2 peaks using Panther and ReViGO [34, 35]. Since 9754 peaks were obtained in the cluster, peak-to-gene annotation would retrieve a large fraction of all genes. To reduce false discovery, we restricted our analysis to gene-proximal peaks located from 5 kb upstream to 1 kb downstream of a given gene. This reduced the overall peak count to only ~1400 peaks, corresponding to 1224 genes. Significantly enriched GO terms related to the stress response, including “immune system process”, and “inflammatory response” (Fig. 6A; Table S2).

To determine whether the same group of stress-responsive genes might upregulate in *rd1* rods, we intersected the 1224 tethering-specific cluster C2 genes with the RNA-seq data generated by Jiang et al., but observed no systematic change in the expression of these genes (Fig. 6B). Moreover, we also examined the viability of transfected cells using flow cytometry, but did not observe any elevation in LA or Lbr expressing rods (Fig. 6C), nor did stress-responsive transcripts upregulate in our Multi-seq dataset (Fig. 6D, E). Nonetheless, a few cluster C2 genes exhibited notable increases in accessibility, including the putative tumor suppressor *Tusc1* (Fig. 6F). Novel peaks were also seen at the *Ccl3* and *Ccl4* chemokine genes (Fig. S11A), as well as the interferon activated gene *Ifi204* and the *Cd68* surface marker (Fig. S11B, C)—both of which were shown to become acutely accessible in a light damage model of retinal degeneration [36]. We also observed novel tethering-specific peaks at the immediate early gene *Nab2* (Fig. 6G). Interestingly, *Nab2* has been shown to upregulate during cone degeneration [37]. Taken together, these data suggest that heterochromatin tethering might ‘poise’ regulatory elements to facilitate the stress response, but that additional steps are necessary for full gene activation.

## Discussion

The rod photoreceptors of nocturnal animals are perhaps the only eukaryotic cells that normally function without heterochromatin tethering. Here, we report that LA upregulates in the *rd1* model of retinitis pigmentosa. Since LA/LC upregulates in two other degenerative models [10, 12], and since *Pde6b* does not directly regulate gene expression, we conclude that *Lmna* upregulation is likely a general response to degeneration.

**Fig. 7:**
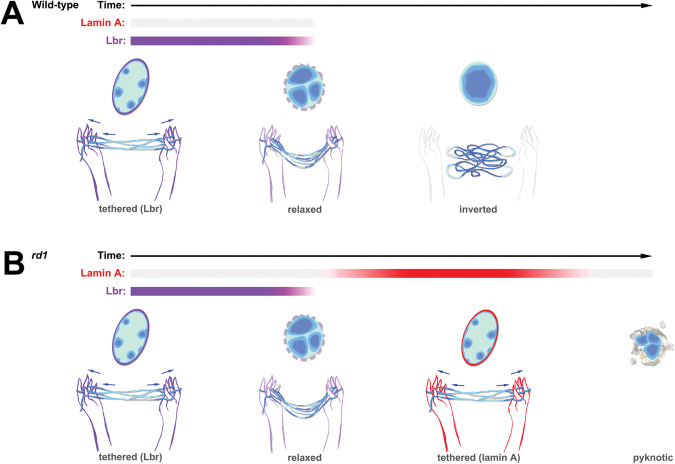
Cat’s cradle model for tethering-dependent effects on genome accessibility. **A** During the differentiation of wild-type rods, the tethering of heterochromatin (dark blue) by Lbr (purple) stretches the chromosomes, promoting accessibility in the A-compartment (cyan). Over time, Lbr expression is downregulated leading to chromatin relaxation, and finally chromatin inversion. As the chromatin relaxes, accessibility decreases. **B** During the differentiation of *rd1* rods, lamin A (red) upregulates at the onset of tissue damage. The prolongation of tethering promotes accessibility at genomic regions that would normally be decommissioned. *Rd1* rods downregulate lamin A prior to cell death.

How does LA upregulation affect the photoreceptor genome? Previous studies suggested that the absence of tethering leads to a strikingly ‘closed’ accessibility signature [11, 38]. Perhaps counterintuitively, our data suggest that tethering the heterochromatic B-compartment at the nuclear periphery mainly affects accessibility within the euchromatic A-compartment. Acting like the fingers in a “cat’s cradle”, heterochromatin tethering might be important for disentangling and segregating B compartment TADs away from the A compartment (Fig. 7). Alternatively, tethering might provide tensile force to chromosomes that could facilitate gene expression. *LMNA* mutations have accordingly been shown to have extensive effects on genome accessibility in other contexts [39].

Based on genome modeling, The Solovei and Mirny labs predicted that the introduction of heterochromatin tethering in fully inverted rods would fail to restore conventional architecture [16]. Results from *SCA7* and *Casz1* mutant mice, in which LA/LC upregulates in mature rods agree with this prediction [12, 16]. By contrast, the upregulation of LA in *rd1* rods occurs prior to full inversion. Nonetheless, since tethering mainly induced novel peaks within the A-compartment, we predict that LA upregulation might have similar effects on genome accessibility during the degeneration of mature rods with fully inverted architecture.

### Heterochromatin tethers are permissive—but not instructive—for gene expression

We found that LA/Lbr both increased genome accessibility similarly—mainly at distal intergenic regions. Focusing on cluster C2 peaks, we found that Ctcf footprints were increased, suggesting that these loci might be bona fide regulatory elements that are normally decommissioned in inverted rods. Accordingly, we found that many of these loci exhibited Ctcf occupancy in murine ES cells. While the accessibility signatures of LA/Lbr were similar, they differed in footprint enrichment profiles and elicited subtle but divergent changes in gene expression, in agreement with previous research [10]. Moreover, morphometric analysis of heterochromatin tethering revealed that LA/Lbr had different effects on chromocenters.

In the literature, LA/Lbr are thought to interact with ‘lamina associated domains’ that are characterized by localization within the B-compartment, and enrichment for heterochromatic marks. As our transfection paradigm only permitted the purification of small numbers of cells, we focused on methods such as scRNA-seq and ATAC-seq. However, ATAC-seq would be unlikely to identify elements directly bound by LA/Lbr. In the future, it will be important to use a battery of ‘omics’ and microscopic approaches in order to determine how the genome interacts with LA/Lbr, and to perform more extensive time coursing and replication.

One alternative possibility is that the increased accessibility observed in tethered rod datasets might arise if samples were contaminated with non-rod cells. However, we disfavor this interpretation. First, in our ATAC-seq experiments, we found that cell-type-specific marker genes exhibited equivalent accessibility when compared to control rod datasets. Second, most of the observed novel peaks were distal to genes. Third, even for gene-proximal peaks, GO terms were mainly associated with the stress response rather than cell fate, suggesting that changes in cell composition are unlikely to account for the novel peaks.

Another alternative interpretation is that the observed accessibility signature might be a by-product of toxicity introduced by construct overexpression. Again, we disfavor this scenario. First, while LA/LC overexpression has been associated with toxicity, these effects are often linked to mitotic catastrophe or nuclear rupture, which are mitigated in non-motile post-mitotic rods. We harvested rods at least 6 weeks after transfection, and observed no effect on cell viability, suggesting that LA was well-tolerated over the long-term. Second, similar changes in accessibility were observed when rods were transfected with Lbr, which has been shown to be well-tolerated in transgenic mice [9, 10]. Third, LA/Lbr are both expressed in the rods of various vertebrates [10].

### Lamin A reorganizes the nucleus during degeneration

ATAC-seq has been recently used to characterize degenerating retinas in age-related macular degeneration and murine light damage models, revealing a marked *decrease* in genome accessibility [36, 40]. Examination of RNA-seq data from the light damage model revealed that *Lmna* was similarly upregulated by ~10–20-fold—both at 6 h and one day post-injury, but not at 3 days [36]. While the reported decrease in genomic accessibility thus conflicts with our observations, the ATAC-seq data from the above studies were generated using whole retinas, whereas we studied sorted rods. Moreover, we note that several genes that were reported to become accessible upon light damage, including *Ccl4, Ifi204*, and *Cd68*, similarly became accessible upon heterochromatin tethering via LA/Lbr (Fig. S11). Luu et al. also reported that light damage increased accessibility at distal intergenic regions, in accordance with our observations.

Elsewhere in the CNS, changes in nuclear lamins have previously been linked to neurodegeneration. For example, alterations in the expression and integrity of B-type lamins have been documented in tauopathies and Alzheimer’s disease [41, 42]. A potential linkage between LA and photoreceptor degeneration nonetheless seemed unlikely, given that the expression of the *LA* splice variant is usually suppressed in neurons [43], and has been repeatedly shown to be absent in rods [10–13]. However, LA was recently found to upregulate in hippocampal neurons in Alzheimer’s disease [44]. Indeed, *Lmna* upregulation was recently linked to tissue damage in a variety of other organs [45], although the responsible regulatory mechanisms have not yet been defined.

What might be the purpose of upregulating LA in response to pathology? Tethering heterochromatin via LA/Lbr transfection appears to ‘poise’ the regulatory elements of stress-responsive genes. However, the limited effect on transcription suggests that LA upregulation may serve additional purposes. One possibility is that heterochromatin tethering may be important for facilitating DNA repair. Indeed, previous studies have shown that DNA repair is inefficient in inverted rod photoreceptors, and this inefficiency is ameliorated via transgenic misexpression of Lbr [46, 47]. Given the well-documented linkage between LA/LC and DNA repair [48], it would be interesting to test whether LA upregulation improves the efficiency of DNA repair even further.

## Methods

### Animals

Animal work was conducted according to the guidelines of the Canadian Council on Animal Care and the Animal Care and Veterinary Service at uOttawa using ethical protocols OHRI-2856 and OHRI-2867. CD1 mice were obtained from Charles River Laboratories. *C57BL/6J* and *rd1* (C57BL/6J-*Pde6b*^*rd1-2J*^/J; strain# 004766) mice were obtained from Jackson Laboratories and maintained as homozygous stocks. Animals of both sexes were used throughout the study.

### DNA constructs

pCIG2 and pCIG2 Lamin A were previously described [12, 49]. A pCIG2 Lbr plasmid was generated by PCR amplifying Lbr from pMSCV-Flag-Lbr, generously provided by Peter Gaines [50] in order to remove the Flag tag. Primers were Lbr XhoI F: 5′-CACACTCGAGATGCCAAGTAGGAAGTTTGTTG-3′ and Lbr EcoRI R: 5′-CACAGAATTCTCAGTAAATGTAGGGGAATATG-3′. To mark rod photoreceptors, we utilized pRho-DsRed generously provided by Connie Cepko (Addgene #11156) [30]. Stable cell lines were generated using pBABE-puro-GFP-wt-lamin A (Addgene #17662) and pBABE-puro-GFP-Progerin (Addgene #17663) plasmids, generously shared by the Tom Misteli lab [51].

### Electroporation

In vivo retinal electroporations were performed as described previously [12, 30]. Briefly, P0 pups were anesthetized on ice, and an incision was made into the eyelid to expose the orbit of the eye. Plasmid DNA (2 µg/µl) was mixed with Fast Green dye and injected subretinally, using a Femtojet microinjector (Eppendorf) and pulled borosilicate needles (Drummond). Pups were placed into an incubator to re-warm, and then replaced into the home cage.

### Flow cytometry

Adult retinas were dissected and placed in StemPro Accutase (Gibco) for 30 min at 37 °C. Cells were triturated manually, incubated with Dapi as a viability marker, and then sorted by the OHRI Flow Cytometry and Cell Sorting Facility using a Beckman Coulter MoFlo XDP.

### Immunohistochemistry and microscopy

Retinas were processed for immunohistochemistry as previously described [12, 52]. We used the following primary antibodies: Nr2e3 (PNR: R&D Systems PP-H7223-00), lamin A (Fortis A303-433A), Lamin A/C (Harald Herrmann Lab), Lbr (Monika Zwerger and Heinrich Leonhardt). Hoechst 33342 (Tocris NB5117) and Alexa Fluor-568-conjugated peanut agglutin (Molecular Probes L32458) were applied along with the primary antibodies.

Images were acquired using Zeiss LSM880 or LSM900 confocal microscopes with Airyscan detectors. All images presented in the paper are from individual Z-planes, and all level transformations were linear. Images were processed using Zen (Zeiss), Fiji (ImageJ), and Adobe Photoshop (Adobe) software.

### Cell culture and western blot

Cell culture and western blotting were performed as previously described [12, 52]. See above for antibody information. Stable cell lines expressing pBABE-puro-GFP-wt-lamin A, pBABE-puro-GFP-Progerin, or GFP control plasmids were generated by transfecting 293 cells (EcoPack2 cell line, Takara) with plasmids and selecting with puromycin (Bio Basic). Cell lines were monitored for mycoplasm, and were additionally treated periodically with BM-cyclin antibiotics (Sigma).

### Nuclear morphometric analysis

Single 8-bit Airyscan Z planes were acquired using fixed confocal settings. Densitometry measurements were performed manually and unblinded using ImageJ and Fiji software [53]. Nuclei were selected for analysis solely on the basis of Nr2e3 expression (Fig. 2) or GFP expression (Fig. 3), without additional exclusion criteria (e.g., GFP intensity, size, shape). The “Freehand Selection” tool was first used to measure mean pixel intensity of each selected nucleus. Then, perimeters were traced along the margin of the visible Hoechst signal using the “Freehand Line” tool, and the mean pixel intensity at the nuclear perimeter was measured. This measurement was divided by the mean pixel intensity of the entire nucleus in order to normalize against cell-to-cell or image-to-image variations in intensity. For the chromocenter midpoint/lamina measure, the distance between the centroid of each chromocenter within a given nucleus and the nuclear periphery was measured (non-cumulatively) using the “Straight Line” tool. Plot profiles for the perimeter intensity were generated using the “Freehand Line” tool in concert with the “Plot Profiles” feature in ImageJ. Plot profiles for transect intensity measurements were performed using “Straight Line” tool with the “Plot Profiles” feature. Cumulative plot profile intensity plots were generated using Graphpad Prism (Graphpad Inc.).

### Statistics

Statistical analyses for count and measurement data were performed using Microsoft Excel and GraphPad Prism software. No blinding/randomization was performed. n-values refer to biological replicates (independent experiments or animals as indicated in the text and figure legends). For cell counting, we aimed for a minimum of 3 biological replicates (30 cells each as technical replicates), and performed one-way ANOVAs, with Tukey’s post-hoc test. We did not perform statistical analyses to predetermine sample sizes. ANOVAs passed tests for normality (Shapiro-Wilk test) and variance (Brown-Forsythe test). All error bars are mean ± SEM. We did not exclude any datapoints in this study, with the exception of cells that did not meet quality control standards in our scRNA-seq analyses (see below).

### ATAC-seq

ATAC-seq data were generated following Buenrostro et al. [54]. Briefly, 50,000 flow-sorted cells were lysed in cold lysis buffer (10 mM Tris-Cl, pH 7.4, 10 mM NaCl, 3 mM MgCl_2_, and 0.1% IGEPAL CA-630). Lysed nuclei were tagmented using 6.5 µl of TDE1 transposase from the Nextera DNA Flex Library kit (Illumina). Samples were purified using Zymo-Spin IC columns (Zymo), and libraries constructed according to the Nextera workflow. Libraries were cleaned up using the AMPure XP kit (Beckman Coulter). PE 150 sequencing was performed using the NextSeq 500 platform to a read-depth of 25–35 million reads per sample.

### Multi-seq

After flow cytometric sorting, cells were barcoded with ‘anchor’ and ‘co-anchor’ lipid-modified oligonucleotides generously provided by the Zev Gartner lab [55]. Barcode oligonucleotides were purchased from Integrated DNA Technologies as follows. Barcode 1: F: 5′- CCTTGGCACCCGAGAATTCCAGGAGAAGAAAAAAAAAAAAAAAAAAAAAAAAAAAAAAA-3′; Barcode 2: F: 5′- CCTTGGCACCCGAGAATTCCACCACAATGAGAAAAAAAAAAAAAAAAAAAAAAAAAAAAAAA-3′; Barcode 3: F: 5′- CCTTGGCACCCGAGAATTCCATGAGACCTAAAAAAAAAAAAAAAAAAAAAAAAAAAAAAA-3′.

Each replicate was incubated with barcode oligonucleotides for 10 min. Cells were pelleted and washed 3 times with PBS. Replicates were pooled and processed in a single 10X Genomics Chromium run. Expression library FASTQs were processed using CellRanger (10X Genomics).

### Bioinformatics—ATAC-seq

ATAC-seq Fastq files were processed via Fastq Groomer [56] and Trimmomatic [57], and then mapped to the mm10 genome using Bowtie2 [58]. Summit and narrowpeak calling was performed with Macs2 [59], and we used GREAT [60] for peak-to-gene annotation. GO terms analysis was performed using Panther [34, 61] followed by ReViGO [35]. ATAC-seq histograms were generated using Seqplots [62].

Sorted rod and double-sorted green cone ATAC-seq data and narrowpeak files (mm10) were obtained from Hughes et al. [11] (GSE83312). Ctcf ChIP-seq data (ENCSR343RKY) generated by the Bing Ren lab [32] were obtained from the ENCODE Consortium [63, 64]. Additional ChIP-seq and cut&run-seq datasets were obtained from the Jeremy Nathans lab [33] (GSE72550), or the Epstein/Poleshko labs [8] (GSE180006).

For comparison with compartment data generated by Falk et al. [16] and Ctcf ChIP-seq data from ENCODE, ATAC-seq data were re-mapped to the mm9 genome as per above, except that we used Cutadapt for adapter trimming.

Footprinting analysis was performed using TOBIAS. As per the guidelines, we merged peak files together: lamin A with control; Lbr with control. Bindetect was performed using 220 motifs selected from the TRANSFAC database.

### Bioinformatics—RNA-seq

RNA-seq data from sorted rod photoreceptors from P10 *Rd1* mice were obtained from Jiang et al. [24] (GSE183117). Figure 1E presents the bioinformatic data published in the original paper. To visualize *Lmna* transcription and splicing, we re-mapped the data to the mm9 genome using Galaxy [65]. Fastq files were processed via Fastq Groomer [56] and Trimmomatic [57], and then mapped to the mm9 genome using RNA Star [66]. Genome visualization and sashimi plots were generated using IGV [67]. Heatmap in Fig. 6D was generated using Morpheus (https://software.broadinstitute.org/Morpheus). We quantitated differential transcripts using FeatureCounts [68] and DeSeq2 [69].

### Bioinformatics—scRNA-seq

Fastq files were aligned to the mm10 genome using CellRanger version 6.1.2 (Cell Ranger software, 10x Genomics). Output files were filtered and analyzed using Scanpy version 1.9.1 [70] in Python (Python Core Team n.d.). Genes detected in less than 3 cells were removed from the analysis. Low-quality cells (less than 200 genes detected, more than 2500 genes detected or more than 18% of mitochondrial genes) were also excluded. Libraries contained an average of 369,327 reads per cell, for a 98.6% saturation depth. Scrublet version 0.2.2 was used to detect doublets [71]. Replicates were demultiplexed with using the MULTI-seq workflow [55]. To annotate cell types, we trained a deep learning model grounded on previously published retinal single cell expression data [29] using scDeepSort version 1.0 [28]. Mitochondrial gene regression and initial gene expression analysis was performed using scVI-tools version 0.19.0 [72]. The following cell numbers passed quality control filters: pCIG2 control: 264 rods. Lamin A: 316 rods. Lbr: 41 rods. Differential gene expression analyses were performed using MAST version 1.24.0 [73]. Data integration was carried out on PostgreSQL version 14.3 (PostgreSQL Core Team n.d.) and Python’s library Pandas version 1.5.2 (Pandas Core Team n.d.).

### Supplementary information

Supplemental Material

Supplemental Movie 1

Supplemental Movie 2

Supplemental Table S1

Supplemental Table S2

Original Data File

Reproducibility checklist

## Data Availability

ATAC-seq data were uploaded to the GEO repository under accession: GSE240465. scRNA-seq data were uploaded to the GEO repository under accession: GSE240312. Microscopy datasets are available from the corresponding author on request.
