# Supplementary material for: Lamin A upregulation reorganizes the genome during rod photoreceptor degeneration
Source: Cell Death Dis. 2023 Oct 25;14(10):701. doi: 10.1038/s41419-023-06224-x (PMC10600220; doi:10.1038/s41419-023-06224-x)
Supplement: Supplementary file 1 — Supplemental Material [file 41419_2023_6224_MOESM1_ESM.pdf]

Supplemental Information

Lamin A upregulation reorganizes the genome during rod photoreceptor degeneration

Ivana Herrera[1,2], José Alex Lourenço Fernandes[1,2], Khatereh Shir-Mohammadi[1,2], Jasmine Levesque[1,2], Pierre Mattar*[1,2,]

[1]Ottawa Hospital Research Institute (OHRI), Ottawa, ON, K1H 8L6
[2]Department of Cellular and Molecular Medicine, University of Ottawa, Ottawa, ON, K1H 8M5

* Correspondence to: pmattar@ohri.ca

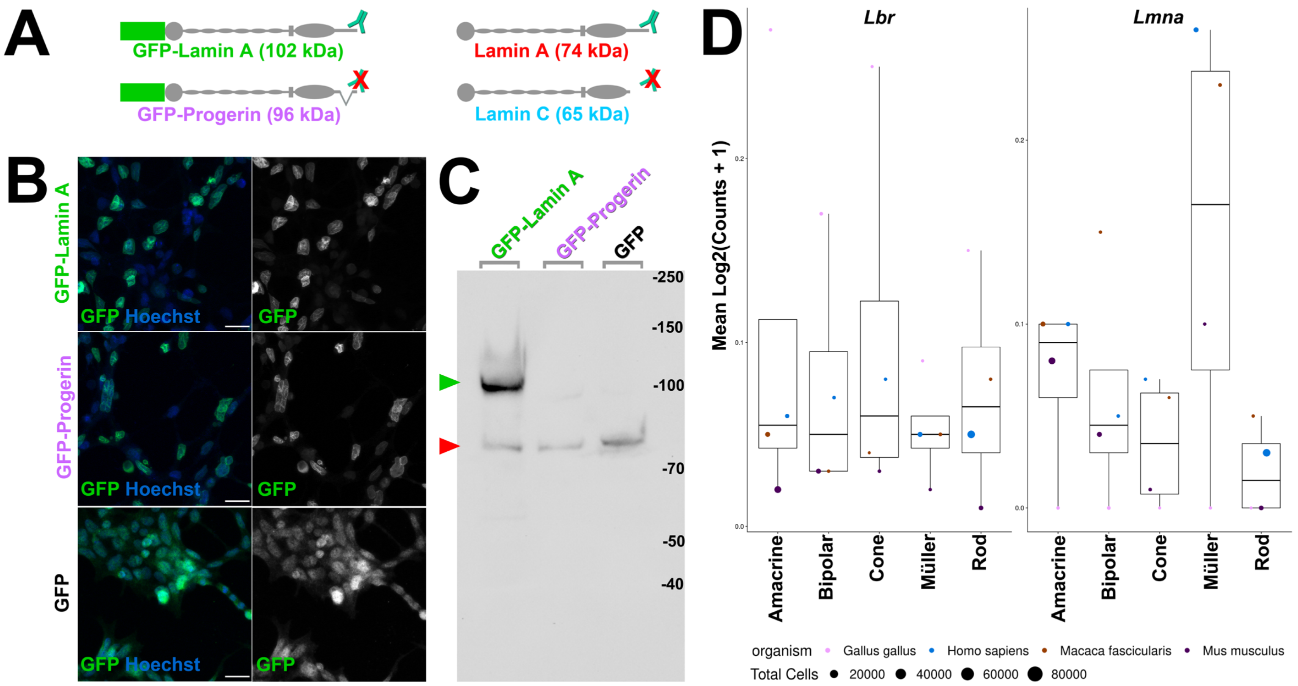

Figure S1. Validation of the lamin A -specific antibody. (A) Schematic of endogenous and exogenous A-type lamin proteins. Lamin A and lamin C are identical from amino acid residues 1-565, but diverge at their respective C-termini. Progerin is a misspliced protein that is identical to lamin A, except that it lacks 50 amino acids (606-656). (B) 293 cell lines stably expressing GFP-lamin A, GFP-progerin, or GFP alone as indicated, with GFP epifluorescence. Scale bar = 20 µm. (C) Western blot of stably transfected 293 cell lines expressing GFP-lamin A, GFP-progerin, or GFP alone. Green arrowhead indicates GFP-lamin A band. Red arrowhead indicates endogenous lamin A. 293 cells endogenously express lamin C (~65 kDa), but the antibody fails to detect the protein in the western blot. (D) scRNA-seq expression data for *Lbr* and *Lmna* obtained from chick (*Gallus gallus*), human (*Homo sapiens*), macaque (*Macaca fascicularis*), or mouse (*Mus musculus*) as indicated. The plot was generated using the *Plae* resource[1].

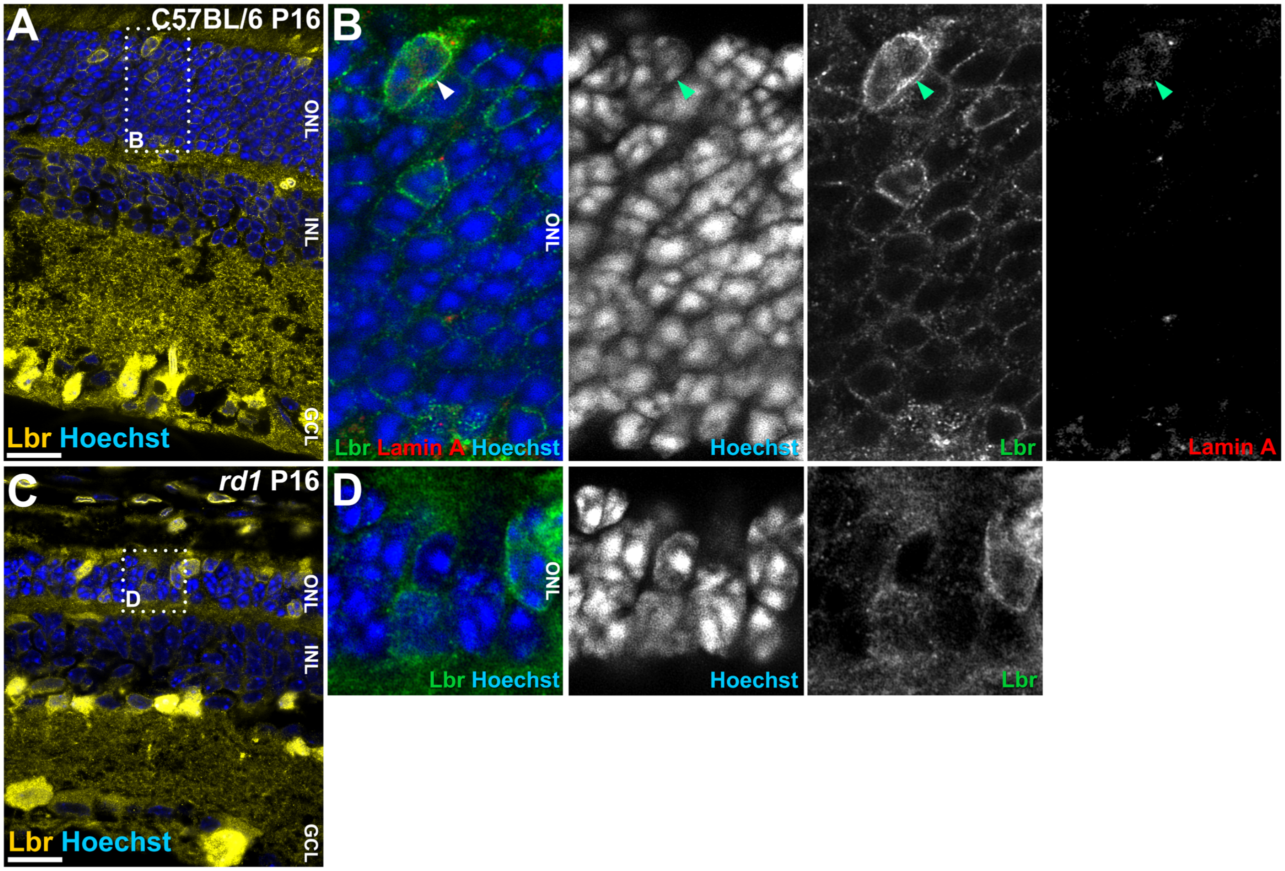

Figure S2. Lbr expression in the degenerating *rd1* mutant. (A-D) Airyscan confocal microscopy of P16 wild-type C57BL/6 (A) or *rd1* mutant retinas (C) with an Lbr-specific antibody (yellow). The retina was also counterstained with DNA dye Hoechst 33342 (blue). Boxed regions indicate the areas shown in the insets. (B, D) Inset regions showing individual color channels, including lamin A for C57BL/6. Arrowheads indicate cone photoreceptors. Scale bars = 10 μm.

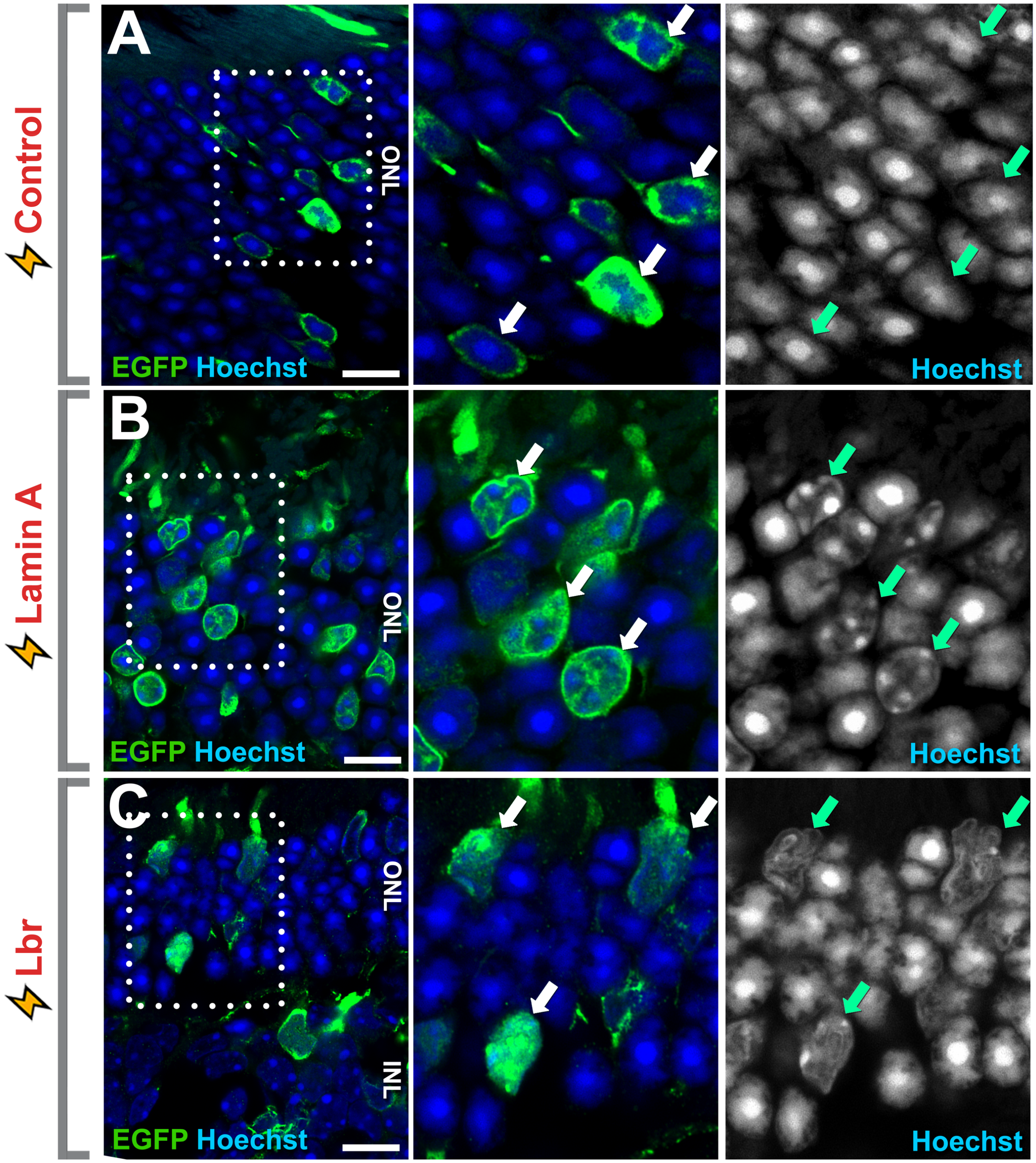

Figure S3. Examples of heterochromatin tethering induced by in vivo electroporation. (A-C) Airyscan confocal imaging of retinas transfected with GFP-expressing empty vector control (A), LA (B), or Lbr (C) expression constructs, and harvested after 6 weeks. Arrows mark transfected EGFP+ cells Scale bars = 10 µm.

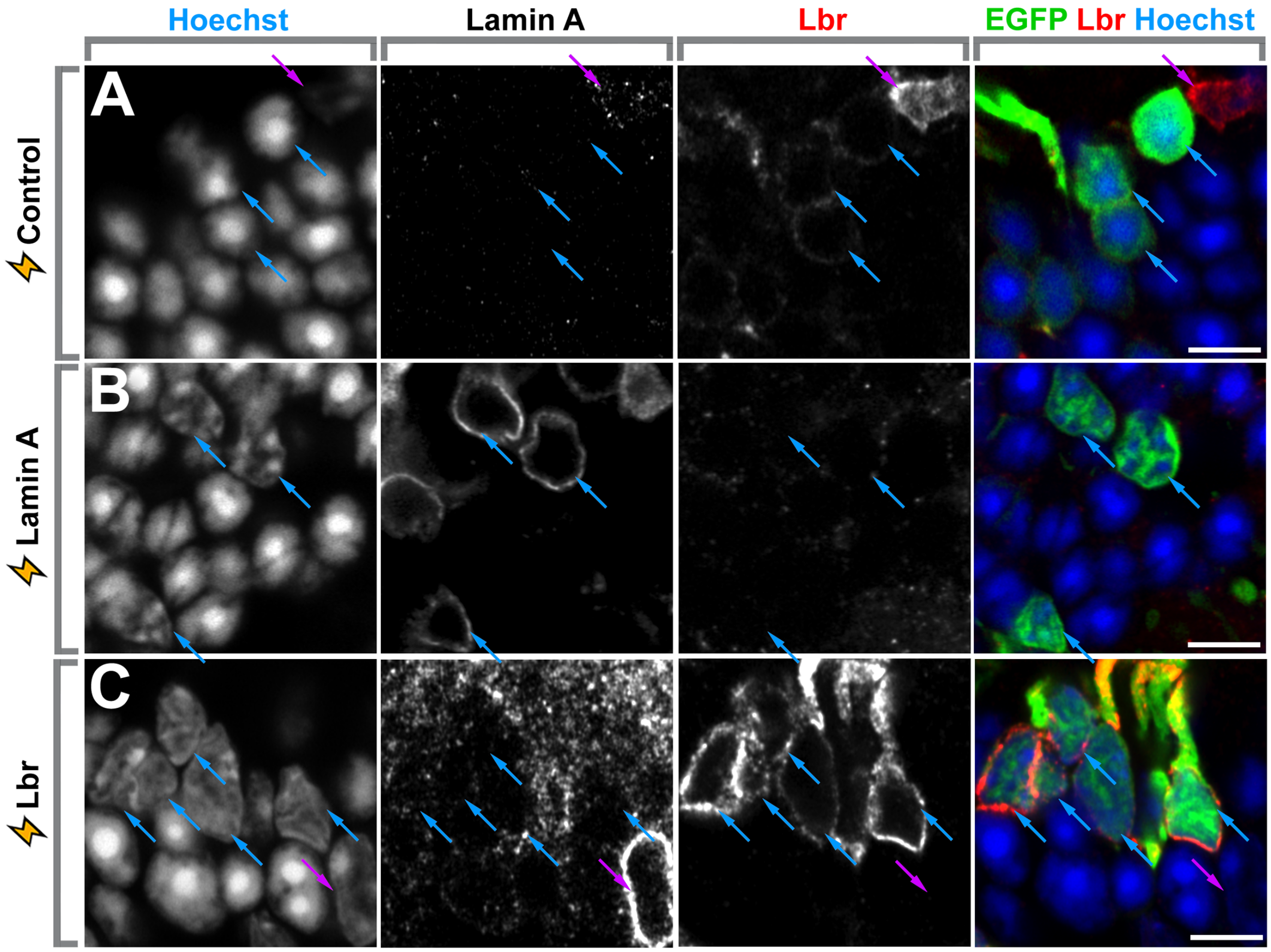

Figure S4. Lack of reciprocal upregulation upon lamin A or Lbr misexpression. (A-C) Airyscan confocal imaging of retinas stained transfected with GFP-expressing empty vector control (A), lamin A (B), or Lbr (C) expression constructs, and harvested after 6 weeks. Retinas were stained for lamin A, Lbr, and Hoechst. Blue arrows mark transfected EGFP+ cells. Magenta arrows indicate cones. Scale bars = 5 µm.

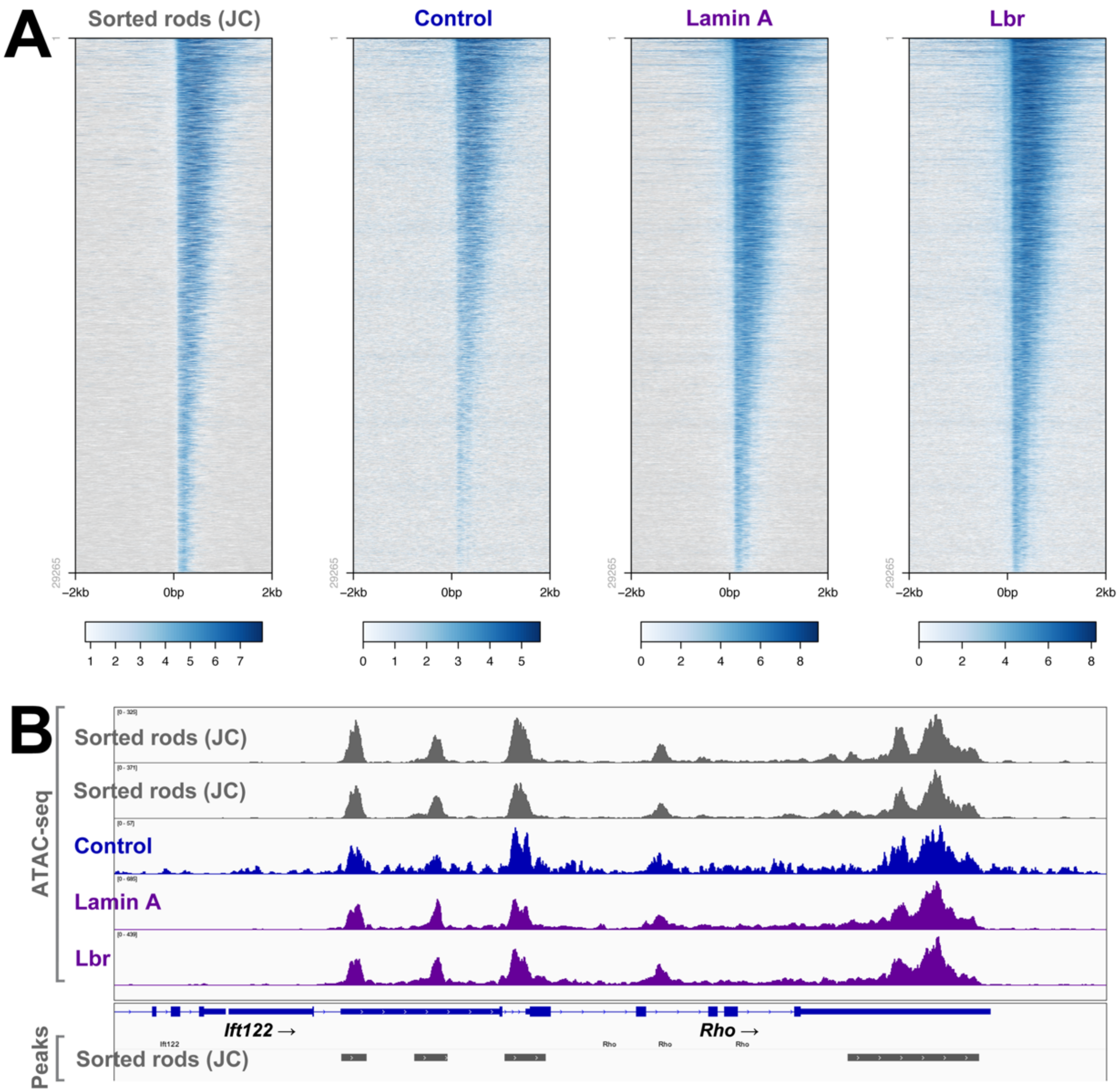

Figure S5. Comparison of control and tethered rod datasets versus previously published rod-specific ATAC-seq data. (A) Alignment of ATAC-seq data from sorted rods transfected with GFP control, lamin A, or Lbr expression constructs compared against previously published data from the Joe Corbo lab (JC) as indicated. Peaks are centered on rod-specific peaks (narrowpeaks) published by Hughes et al.[2] (B) Visualization of previously published ATAC-seq tracks and called peaks previously generated by Hughes et al. (JC), in comparison with ATAC-seq tracks generated from GFP control, lamin A, or Lbr -transfected rods at the *Rhodopsin* locus. Tracks are plotted on different scales.

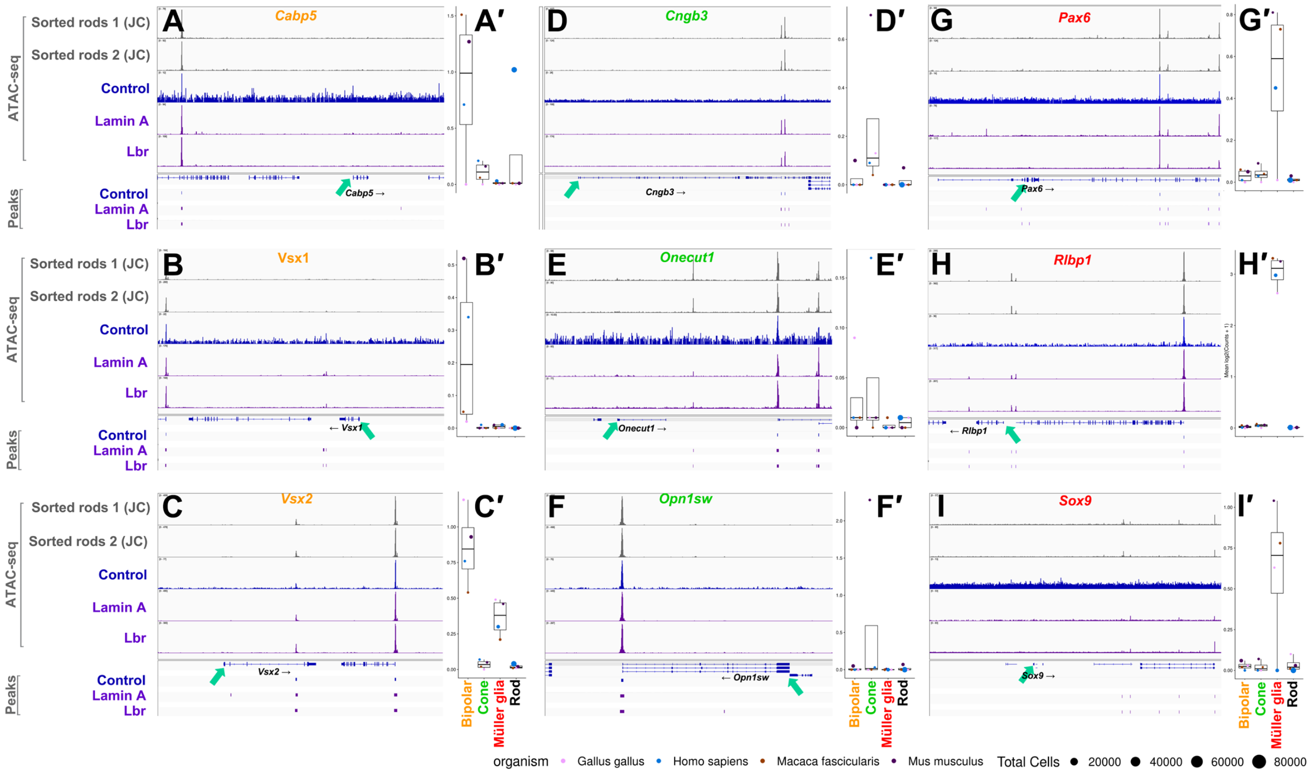

Figure S6. Lack of accessibility at selected cell-type-specific marker genes. (A-I) Visualization of previously published ATAC-seq tracks previously generated by Hughes et al. (JC), in comparison with ATAC-seq tracks and called peaks generated from GFP control, LA, or Lbr -transfected rods at bipolar (A-C), cone (D-F), or Müller glia (E-F) marker genes. ATAC-seq tracks were plotted on different scales. Arrows indicate the transcription start sites for each marker gene. (A′-I′) Comparison of marker gene expression in annotated bipolars, cones, Müller glia, and rods generated using the *Plae* scRNA-seq resource[1].

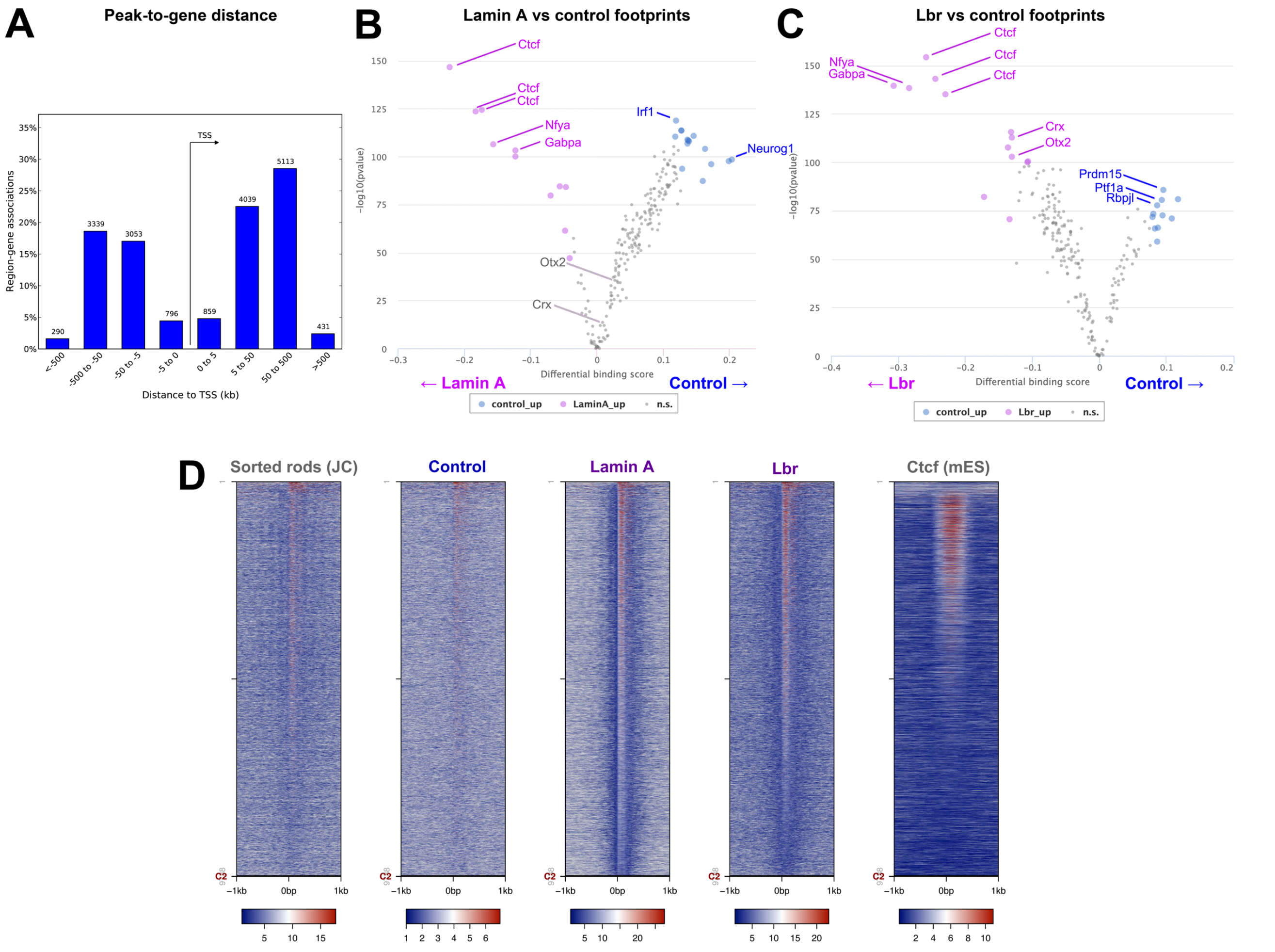

Figure S7. Characterizing novel tethering-specific accessible sites. (A) Location of tethering-specific cluster C2 peaks in relation to the transcription start site (TSS) of adjacent genes. (B, C) Motif enrichment in ATAC-seq footprints comparing LA versus GFP control (B) or Lbr versus control (C) ATAC-seq datasets. (D) Alignment of ATAC-seq data from sorted rods transfected with control, LA, or Lbr expression constructs compared against previously published data from the Joe Corbo lab (JC)[2], and Ctcf ChIP-seq data from embryonic stem cells generated by the Encode Consortium. Plots are centered on tethering-specific cluster C2 peak summits (see Fig. 4D).

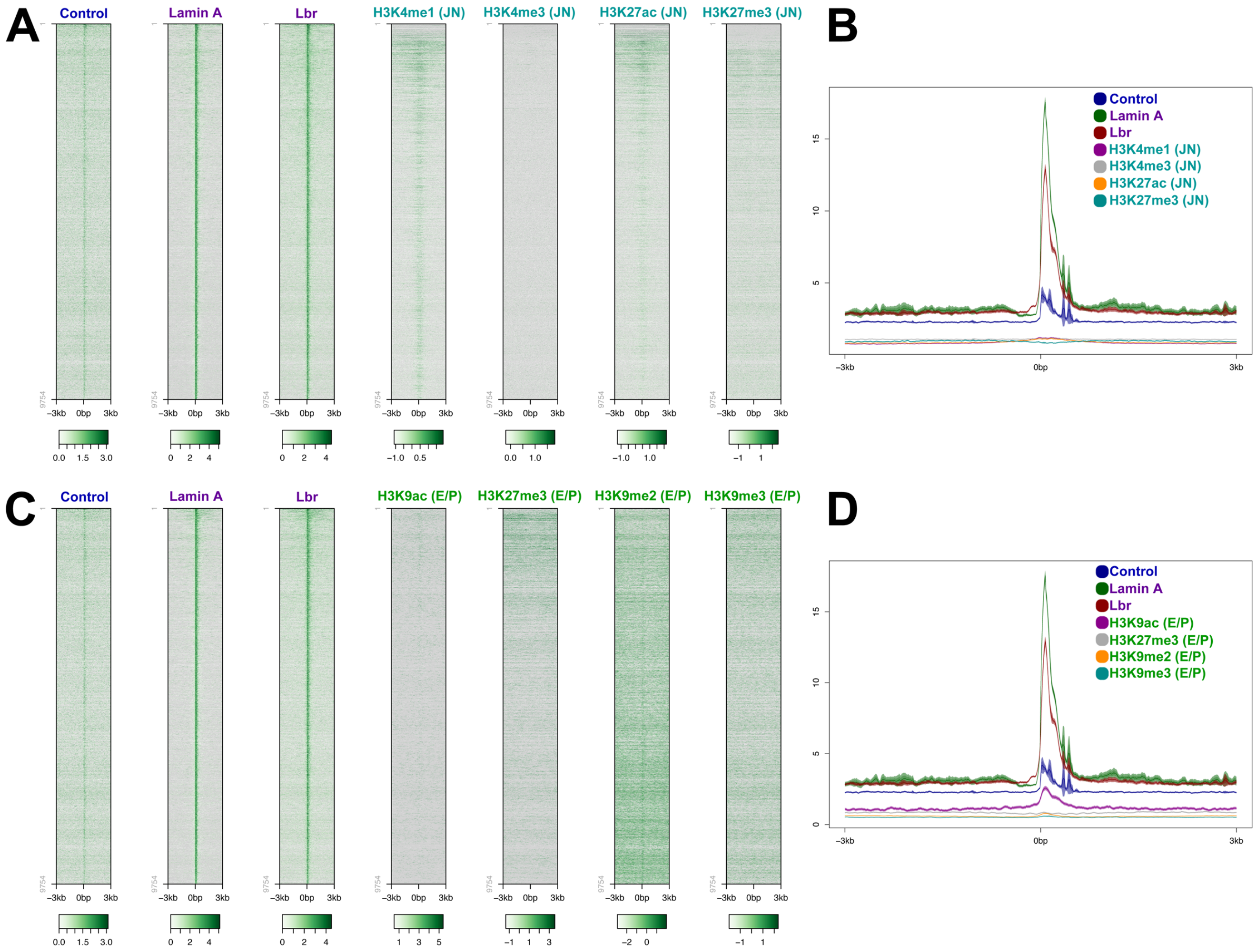

Figure S8. Comparison of control and tethered rod ATAC-seq datasets versus previously published rod-specific ChIP-seq and cut&run-seq data. (A, B) Alignment of ATAC-seq data from sorted rods transfected with GFP control, lamin A, or Lbr expression constructs compared against previously published ChIP-seq data from the Jeremy Nathans lab (JN)[3] as indicated. Plots are centered on tethering-specific cluster C2 peak summits (see Fig. 4D). (C, D) Alignment of ATAC-seq data from sorted rods transfected with GFP control, lamin A, or Lbr expression constructs compared against previously published cut&run-seq data from the Epstein and Poleshko laboratories (E/P)[4] as indicated. Plots are centered on tethering-specific cluster C2 peak summits (see Fig. 4D). Tracks are plotted on different scales.

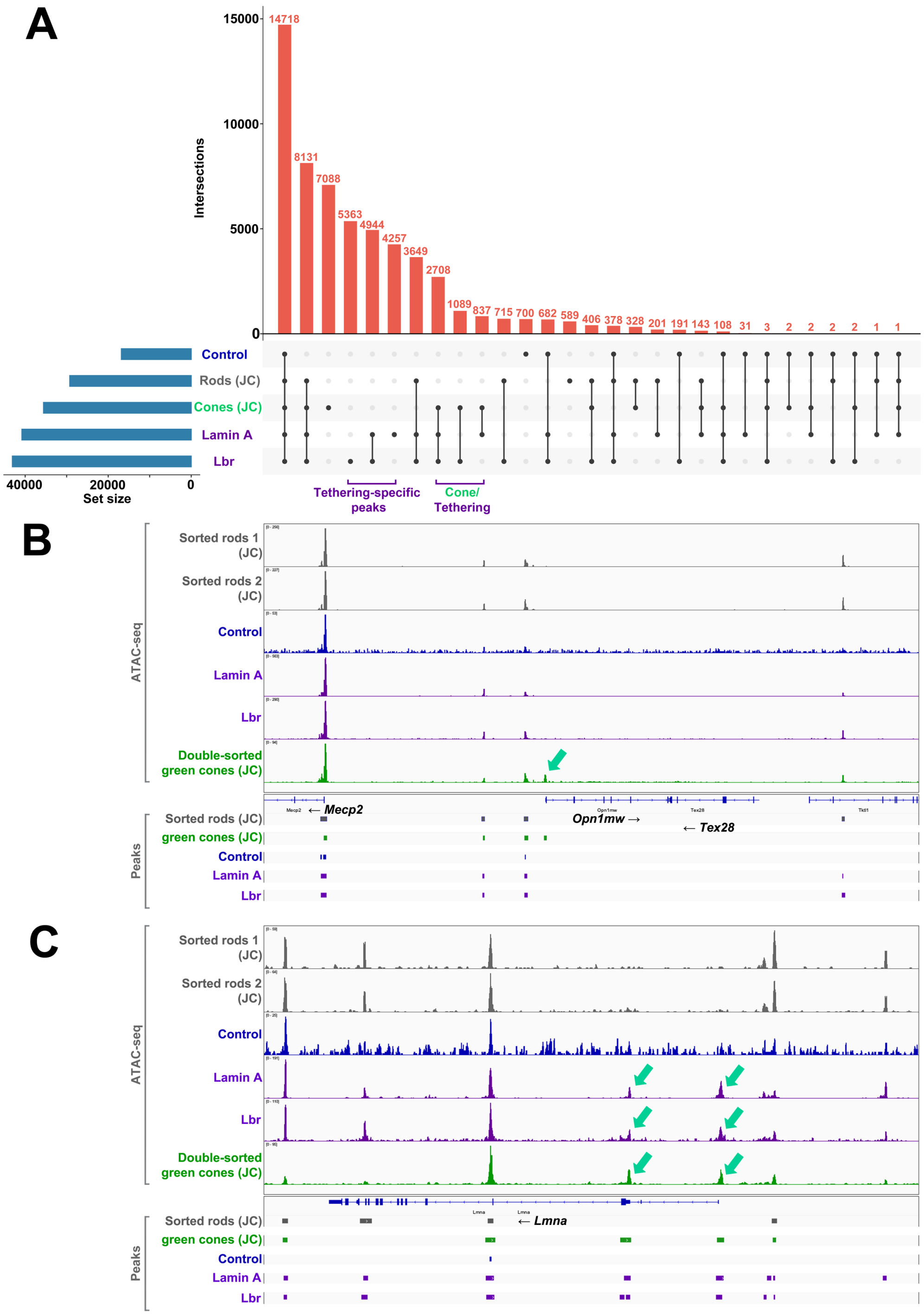

Figure S9. Comparison of ATAC-seq data from control rods, tethered rods, and green cones. (A) Upset plot of of ATAC-seq peak intersections from sorted rods transfected with GFP control, LA, or Lbr expression constructs compared against previously published sorted rod and (double-sorted) green cone data from the Joe Corbo lab (JC)[2] as indicated. (B, C) Comparison of previously published ATAC-seq tracks and called peaks versus ATAC-seq tracks generated from control, LA, or Lbr - transfected rods as indicated, visualizing the cone-specific genes *Opn1mw* (green cone opsin; B) or *Lmna* (C). ATAC-seq tracks are plotted on different scales. Arrows indicate peaks present in cones and/or tethered rods, but not control rods. Tracks are plotted on different scales.

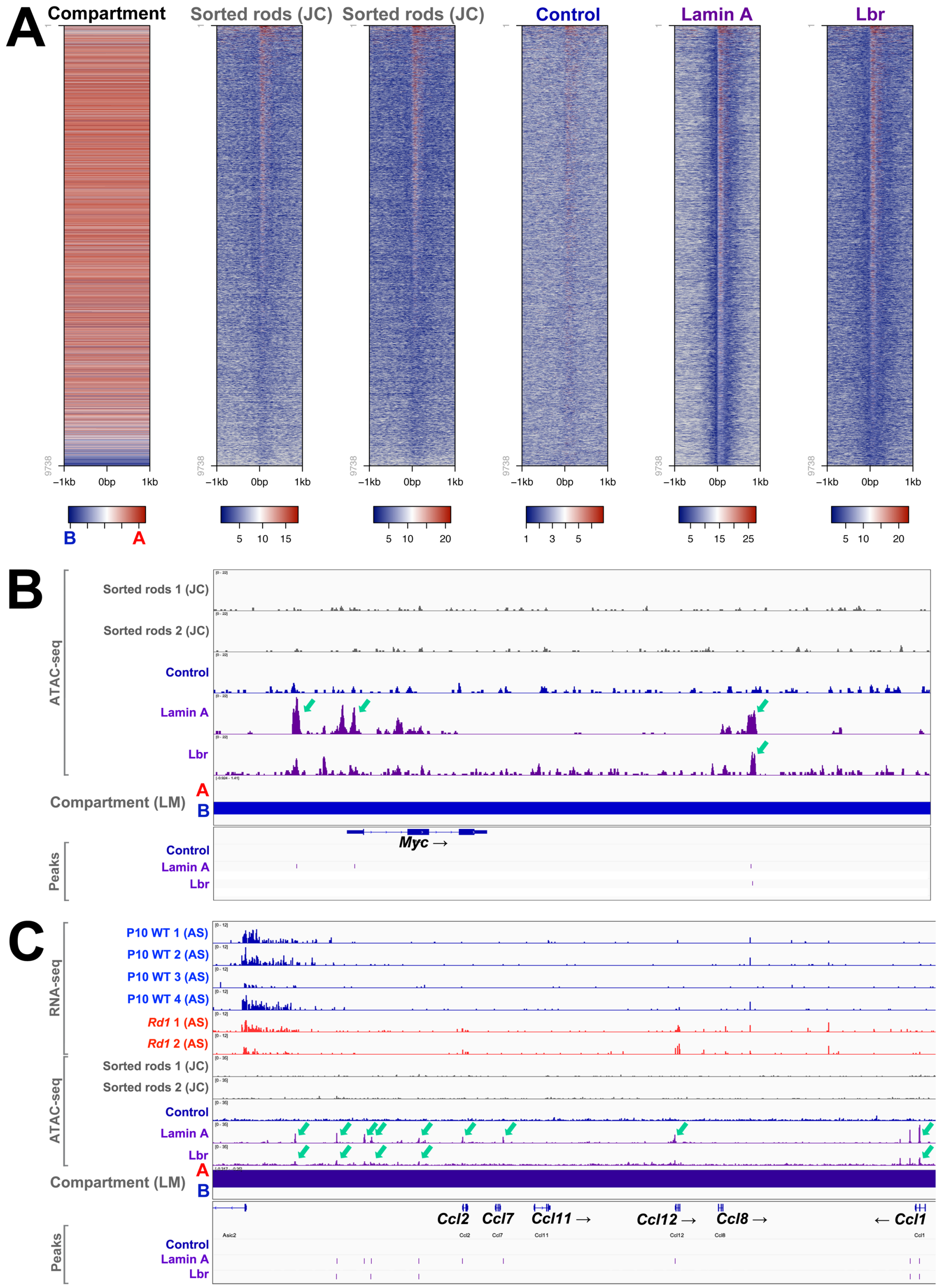

Figure S10. Alteration of genome accessibility in the A versus B -compartments. (A) Alignment of ATAC-seq data from sorted rods transfected with GFP control, LA, or Lbr expression constructs compared against previously published data from the Joe Corbo lab (JC)[2], and compartment data from the Leonid Mirny lab (LM)[5] as indicated. Peaks are centered on tethering-specific cluster C2 peak summits (see Fig. 4D). (B, C) Control vs. *rd1* RNA-seq from the Anand Swaroop lab (AS)[6], ATAC-seq tracks and called peaks from the Joe Corbo lab (JC)[2], and compartment data from the Leonid Mirny lab (LM)[5], compared against ATAC-seq tracks generated from control, LA, or Lbr -transfected rods. B-compartment TADs included the *Myc* (B) and *Ccl2*, *Ccl7*, *Ccl11*, *Ccl12*, *Ccl8*, and *Ccl1* (C) loci. RNA-seq and ATAC-seq tracks were respectively group-autoscaled. Arrows indicate peaks present specifically in tethered rods.

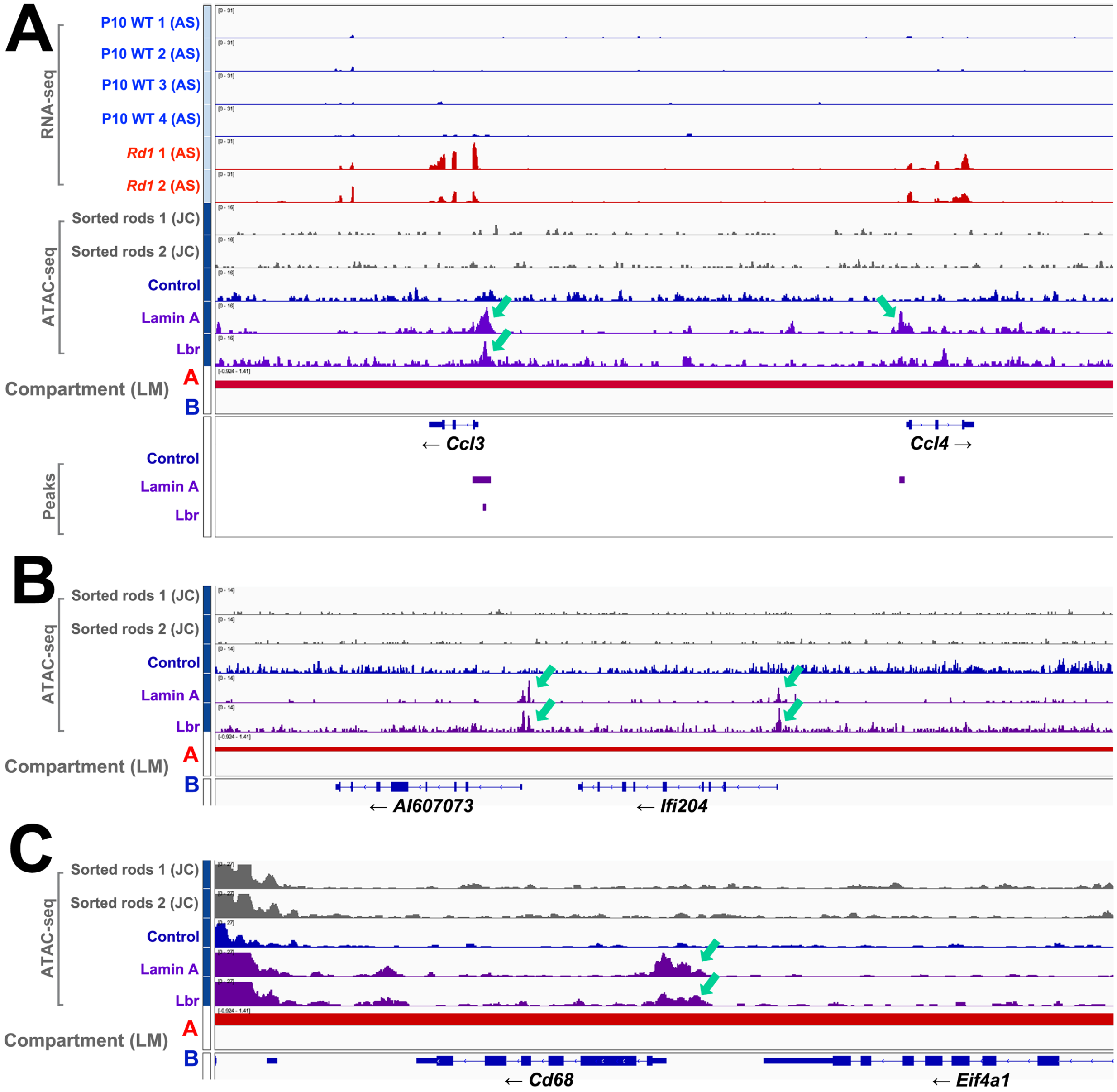

Figure S11. Heterochromatin tethering promotes accessibility at a subset of degeneration-associated genes. (A-C) Control vs. *rd1* RNA-seq from the Anand Swaroop lab (AS)[6], ATAC-seq tracks and called peaks from the Joe Corbo lab (JC)[2], and compartment data from the Leonid Mirny lab (LM)[5], compared against ATAC-seq tracks generated from control, LA, or Lbr -transfected rods as indicated. RNA-seq and ATAC-seq tracks were respectively group-autoscaled. Arrows indicate peaks present specifically in tethered rods, but not control rods.

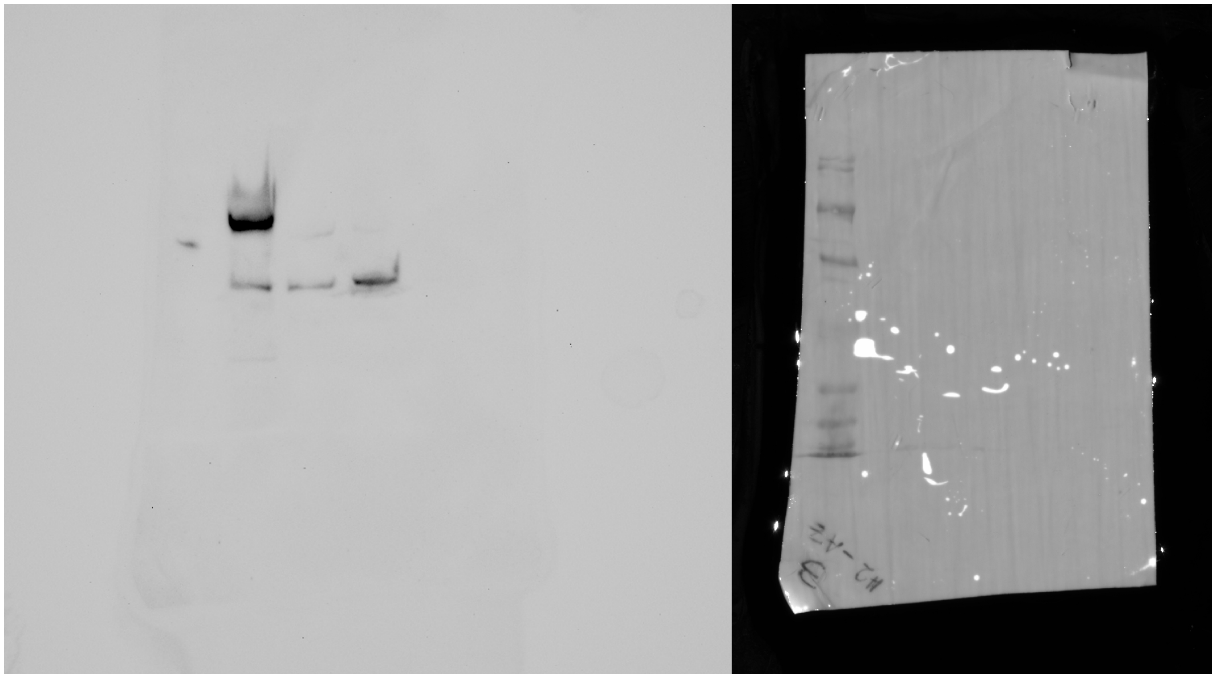

Figure S12. Uncropped scans of western from Fig. S1.

Supplemental Movie 1. Lamin A expression in C57BL6/J control rods. Airyscan confocal microscopy of P16 rd1 retina stained for lamin A (white) and hoechst (blue).

Supplemental Movie 2. Lamin A upregulation in rd1 rods. Airyscan confocal microscopy of P16 rd1 retina stained for lamin A (white), Nr2e3 (green), and hoechst (blue).

Supplemental Table S1. Differentially expressed genes in annotated rod photoreceptors as determined via MAST[7]. Tab 1: Lamin A versus Control. Tab 2: Lbr versus Control.

Supplemental Table S2. Genes and gene ontologies associated with tethering-specific cluster C2 peaks. Peak-to gene annotation was performed using GREAT[8]. Tab 1: peak-to-gene annotation via GREAT. Tab 2: ReViGO analysis for Biological Process (Panther). Tab3: Gene ontology for Biological Process. Tab 4: Gene ontology for Cellular Component. Tab 5: Gene ontology for Molecular Function. Tab 6: Gene ontology for Human Phenotype. Tab 7: Gene ontology for Mouse Phenotype Single KO. Tab 8: Gene ontology for Mouse Phenotype

References

1. Swamy VS, Fufa TD, Hufnagel RB, McGaughey DM. Building the mega single-cell transcriptome ocular meta-atlas. *Gigascience* 2021, **10**(10).

2. Hughes AE, Enright JM, Myers CA, Shen SQ, Corbo JC. Cell Type-Specific Epigenomic Analysis Reveals a Uniquely Closed Chromatin Architecture in Mouse Rod Photoreceptors. *Scientific reports* 2017, **7:** 43184.

3. Mo A, Luo C, Davis FP, Mukamel EA, Henry GL, Nery JR*, et al.* Epigenomic landscapes of retinal rods and cones. *eLife* 2016, **5:** e11613.

4. Smith CL, Lan Y, Jain R, Epstein JA, Poleshko A. Global chromatin relabeling accompanies spatial inversion of chromatin in rod photoreceptors. *Sci Adv* 2021, **7**(39)**:** eabj3035.

5. Falk M, Feodorova Y, Naumova N, Imakaev M, Lajoie BR, Leonhardt H*, et al.* Heterochromatin drives compartmentalization of inverted and conventional nuclei. *Nature* 2019, **570**(7761)**:** 395-399.

6. Jiang K, Mondal AK, Adlakha YK, Gumerson J, Aponte A, Gieser L*, et al.* Multiomics analyses reveal early metabolic imbalance and mitochondrial stress in neonatal photoreceptors leading to cell death in Pde6brd1/rd1 mouse model of retinal degeneration. *Human molecular genetics* 2022, **31**(13)**:** 2137-2154.

7. Finak G, McDavid A, Yajima M, Deng J, Gersuk V, Shalek AK*, et al.* MAST: a flexible statistical framework for assessing transcriptional changes and characterizing heterogeneity in single-cell RNA sequencing data. *Genome biology* 2015, **16:** 278.

8. McLean CY, Bristor D, Hiller M, Clarke SL, Schaar BT, Lowe CB*, et al.* GREAT improves functional interpretation of cis-regulatory regions. *Nature biotechnology* 2010, **28**(5)**:** 495-501.
