# Supplementary figures and images for: Lamin A upregulation reorganizes the genome during rod photoreceptor degeneration
Source: Cell Death Dis. 2023 Oct 25;14(10):701. doi: 10.1038/s41419-023-06224-x (PMC10600220; doi:10.1038/s41419-023-06224-x)

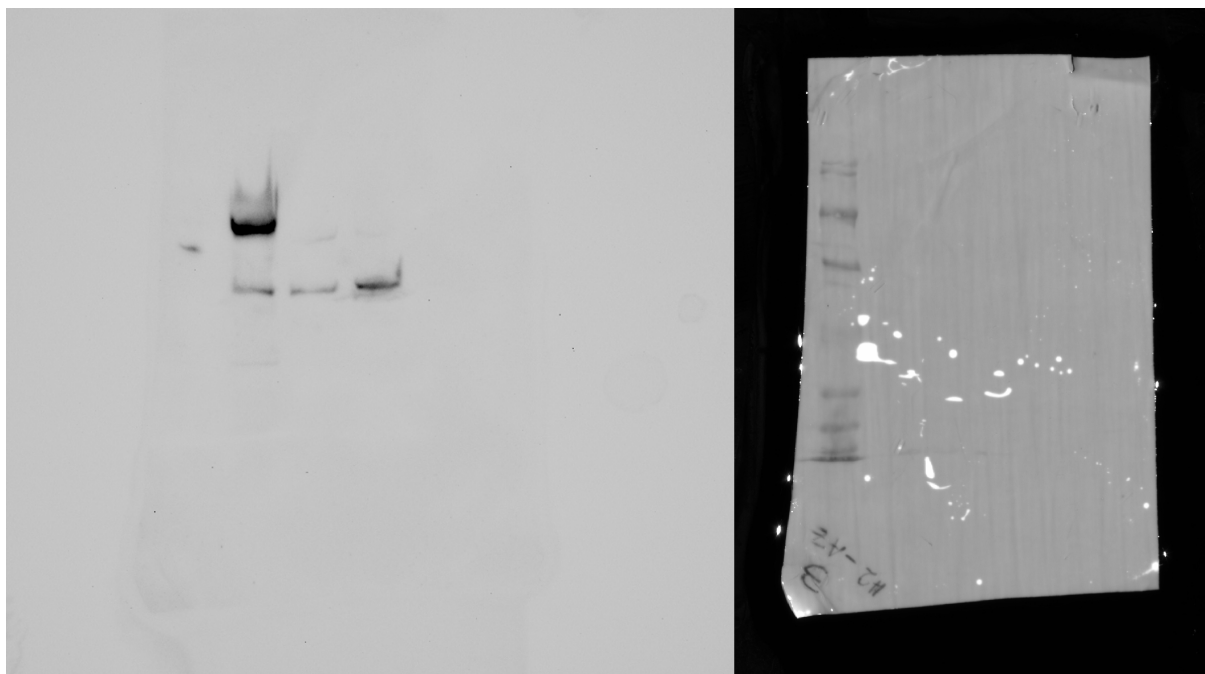

**Figure S12.** Uncropped scans of western from Fig. S1.

Supplement: Supplementary file 6 — Original Data File [file 41419_2023_6224_MOESM6_ESM.pdf]
